# Search for heavy resonances decaying into two Higgs bosons in the $\text {b}{\bar{\text {b}}} \tau ^{+} \tau ^{-}$ final state in proton–proton collisions at $\sqrt{s} = 13\,\text {Te}\hspace{-.08em}\text {V}$

**DOI:** 10.1140/epjc/s10052-026-15699-9

**Published:** 2026-07-30

**Authors:** CMS Collaboration, A. Hayrapetyan, V. Makarenko, A. Tumasyan, W. Adam, L. Benato, T. Bergauer, M. Dragicevic, P. S. Hussain, M. Jeitler, N. Krammer, A. Li, D. Liko, M. Matthewman, J. Schieck, R. Schöfbeck, M. Shooshtari, M. Sonawane, W. Waltenberger, C.-E. Wulz, T. Janssen, H. Kwon, D. Ocampo Henao, T. Van Laer, P. Van Mechelen, J. Bierkens, N. Breugelmans, J. D’Hondt, S. Dansana, A. De Moor, M. Delcourt, F. Heyen, Y. Hong, P. Kashko, S. Lowette, I. Makarenko, D. Müller, S. Tavernier, M. Tytgat, G. P. Van Onsem, S. Van Putte, D. Vannerom, B. Bilin, B. Clerbaux, A. K. Das, I. De Bruyn, G. De Lentdecker, H. Evard, L. Favart, P. Gianneios, A. Khalilzadeh, F. A. Khan, A. Malara, M. A. Shahzad, A. Sharma, L. Thomas, M. Vanden Bemden, C. Vander Velde, P. Vanlaer, F. Zhang, M. De Coen, D. Dobur, C. Giordano, G. Gokbulut, K. Kaspar, D. Kavtaradze, D. Marckx, K. Skovpen, A. M. Tomaru, N. Van Den Bossche, J. van der Linden, J. Vandenbroeck, H. Aarup Petersen, S. Bein, A. Benecke, A. Bethani, G. Bruno, A. Cappati, J. De Favereau De Jeneret, C. Delaere, F. Gameiro Casalinho, A. Giammanco, A. O. Guzel, V. Lemaitre, J. Lidrych, P. Malek, P. Mastrapasqua, S. Turkcapar, G. A. Alves, M. Barroso Ferreira Filho, E. Coelho, C. Hensel, D. Matos Figueiredo, T. Menezes De Oliveira, C. Mora Herrera, P. Rebello Teles, M. Soeiro, E. J. Tonelli Manganote, A. Vilela Pereira, W. L. Aldá Júnior, H. Brandao Malbouisson, W. Carvalho, J. Chinellato, M. Costa Reis, E. M. Da Costa, G. G. Da Silveira, D. De Jesus Damiao, S. Fonseca De Souza, R. Gomes De Souza, S. S. Jesus, T. Laux Kuhn, M. Macedo, K. Mota Amarilo, L. Mundim, H. Nogima, J. P. Pinheiro, A. Santoro, A. Sznajder, M. Thiel, F. Torres Da Silva De Araujo, C. A. Bernardes, L. Calligaris, F. Damas, T. R. Fernandez Perez Tomei, E. M. Gregores, B. Lopes Da Costa, I. Maietto Silverio, P. G. Mercadante, S. F. Novaes, Sandra S. Padula, V. Scheurer, A. Aleksandrov, G. Antchev, P. Danev, R. Hadjiiska, P. Iaydjiev, M. Shopova, G. Sultanov, A. Dimitrov, L. Litov, B. Pavlov, P. Petkov, A. Petrov, S. Keshri, D. Laroze, S. Thakur, W. Brooks, T. Cheng, T. Javaid, L. Wang, L. Yuan, Z. Hu, Z. Liang, J. Liu, X. Wang, H. Yang, G. M. Chen, H. S. Chen, M. Chen, Y. Chen, Q. Hou, X. Hou, F. Iemmi, C. H. Jiang, H. Liao, G. Liu, Z.-A. Liu, J. N. Song, S. Song, J. Tao, C. Wang, J. Wang, H. Zhang, J. Zhao, A. Agapitos, Y. Ban, A. Carvalho Antunes De Oliveira, S. Deng, B. Guo, Q. Guo, C. Jiang, A. Levin, C. Li, Q. Li, Y. Mao, S. Qian, S. J. Qian, X. Qin, C. Quaranta, X. Sun, D. Wang, J. Wang, M. Zhang, Y. Zhao, C. Zhou, S. Yang, Z. You, N. Lu, G. Bauer, Z. Cui, B. Li, H. Wang, K. Yi, J. Zhang, Y. Li, Y. Zhou, Z. Lin, C. Lu, M. Xiao, C. Avila, D. A. Barbosa Trujillo, A. Cabrera, C. Florez, J. Fraga, J. A. Reyes Vega, C. Rendón, M. Rodriguez, A. A. Ruales Barbosa, J. D. Ruiz Alvarez, N. Godinovic, D. Lelas, A. Sculac, M. Kovac, A. Petkovic, T. Sculac, P. Bargassa, V. Brigljevic, B. K. Chitroda, D. Ferencek, K. Jakovcic, A. Starodumov, T. Susa, A. Attikis, K. Christoforou, S. Konstantinou, C. Leonidou, L. Paizanos, F. Ptochos, P. A. Razis, H. Rykaczewski, H. Saka, A. Stepennov, M. Finger, M. Finger, E. Ayala, E. Carrera Jarrin, A. A. Abdelalim, B. El-mahdy, A. Hussein, H. Mohammed, K. Jaffel, M. Kadastik, T. Lange, C. Nielsen, J. Pata, M. Raidal, N. Seeba, L. Tani, E. Brücken, A. Milieva, K. Osterberg, M. Voutilainen, F. Garcia, P. Inkaew, K. T. S. Kallonen, R. Kumar Verma, T. Lampén, K. Lassila-Perini, B. Lehtela, S. Lehti, T. Lindén, N. R. Mancilla Xinto, M. Myllymäki, M. m. Rantanen, S. Saariokari, N. T. Toikka, J. Tuominiemi, N. Bin Norjoharuddeen, H. Kirschenmann, P. Luukka, H. Petrow, M. Besancon, F. Couderc, M. Dejardin, D. Denegri, P. Devouge, J. L. Faure, F. Ferri, P. Gaigne, S. Ganjour, P. Gras, F. Guilloux, G. Hamel de Monchenault, M. Kumar, V. Lohezic, Y. Maidannyk, J. Malcles, F. Orlandi, L. Portales, S. Ronchi, M. Ö. Sahin, P. Simkina, M. Titov, M. Tornago, R. Amella Ranz, F. Beaudette, G. Boldrini, P. Busson, C. Charlot, M. Chiusi, T. D. Cuisset, O. Davignon, A. De Wit, T. Debnath, I. T. Ehle, S. Ghosh, A. Gilbert, R. Granier de Cassagnac, L. Kalipoliti, M. Manoni, M. Nguyen, S. Obraztsov, C. Ochando, R. Salerno, J. B. Sauvan, Y. Sirois, G. Sokmen, Y. Song, L. Urda Gómez, A. Zabi, A. Zghiche, J.-L. Agram, J. Andrea, D. Bloch, J.-M. Brom, E. C. Chabert, C. Collard, G. Coulon, S. Falke, U. Goerlach, R. Haeberle, A.-C. Le Bihan, M. Meena, O. Poncet, G. Saha, A. Savoy-Navarro, P. Vaucelle, A. Di Florio, B. Orzari, D. Amram, S. Beauceron, B. Blancon, G. Boudoul, N. Chanon, D. Contardo, P. Depasse, H. El Mamouni, J. Fay, E. Fillaudeau, S. Gascon, M. Gouzevitch, C. Greenberg, G. Grenier, B. Ille, E. Jourd’Huy, M. Lethuillier, B. Massoteau, L. Mirabito, A. Purohit, M. Vander Donckt, C. Verollet, J. Xiao, G. Adamov, I. Lomidze, Z. Tsamalaidze, V. Botta, S. Consuegra Rodríguez, L. Feld, K. Klein, M. Lipinski, P. Nattland, V. Oppenländer, A. Pauls, D. Pérez Adán, N. Röwert, C. Daumann, S. Diekmann, N. Eich, D. Eliseev, F. Engelke, J. Erdmann, M. Erdmann, B. Fischer, T. Hebbeker, K. Hoepfner, F. Ivone, A. Jung, N. Kumar, M. y. Lee, F. Mausolf, M. Merschmeyer, A. Meyer, A. Pozdnyakov, W. Redjeb, H. Reithler, U. Sarkar, V. Sarkisovi, A. Schmidt, C. Seth, A. Sharma, J. L. Spah, V. Vaulin, S. Zaleski, M. R. Beckers, C. Dziwok, G. Flügge, N. Hoeflich, T. Kress, A. Nowack, O. Pooth, A. Stahl, A. Zotz, A. Abel, M. Aldaya Martin, J. Alimena, S. Amoroso, Y. An, I. Andreev, J. Bach, S. Baxter, H. Becerril Gonzalez, O. Behnke, A. Belvedere, F. Blekman, K. Borras, A. Campbell, S. Chatterjee, L. X. Coll Saravia, G. Eckerlin, D. Eckstein, E. Gallo, A. Geiser, M. Guthoff, A. Hinzmann, L. Jeppe, M. Kasemann, C. Kleinwort, R. Kogler, M. Komm, D. Krücker, W. Lange, D. Leyva Pernia, K.-Y. Lin, K. Lipka, W. Lohmann, J. Malvaso, R. Mankel, I.-A. Melzer-Pellmann, M. Mendizabal Morentin, A. B. Meyer, G. Milella, K. Moral Figueroa, A. Mussgiller, L. P. Nair, J. Niedziela, A. Nürnberg, J. Park, E. Ranken, A. Raspereza, D. Rastorguev, L. Rygaard, M. Scham, S. Schnake, P. Schütze, C. Schwanenberger, D. Schwarz, D. Selivanova, K. Sharko, M. Shchedrolosiev, D. Stafford, M. Torkian, A. Ventura Barroso, R. Walsh, D. Wang, Q. Wang, K. Wichmann, L. Wiens, C. Wissing, Y. Yang, S. Zakharov, A. Zimermmane Castro Santos, A. R. Alves Andrade, M. Antonello, S. Bollweg, M. Bonanomi, L. Ebeling, K. El Morabit, Y. Fischer, M. Frahm, E. Garutti, A. Grohsjean, A. A. Guvenli, J. Haller, D. Hundhausen, G. Kasieczka, P. Keicher, R. Klanner, W. Korcari, T. Kramer, C. c. Kuo, F. Labe, J. Lange, A. Lobanov, J. Matthiesen, L. Moureaux, K. Nikolopoulos, A. Paasch, K. J. Pena Rodriguez, N. Prouvost, B. Raciti, M. Rieger, D. Savoiu, P. Schleper, M. Schröder, J. Schwandt, M. Sommerhalder, H. Stadie, G. Steinbrück, R. Ward, B. Wiederspan, M. Wolf, C. Yede, S. Brommer, A. Brusamolino, E. Butz, Y. M. Chen, T. Chwalek, A. Dierlamm, G. G. Dincer, D. Druzhkin, U. Elicabuk, N. Faltermann, M. Giffels, A. Gottmann, F. Hartmann, M. Horzela, F. Hummer, U. Husemann, J. Kieseler, M. Klute, J. Knolle, R. Kunnilan Muhammed Rafeek, O. Lavoryk, J. M. Lawhorn, S. Maier, A. A. Monsch, M. Mormile, T h. Müller, E. Pfeffer, M. Presilla, G. Quast, K. Rabbertz, B. Regnery, R. Schmieder, N. Shadskiy, I. Shvetsov, H. J. Simonis, L. Sowa, L. Stockmeier, K. Tauqeer, M. Toms, B. Topko, N. Trevisani, C. Verstege, T. Voigtländer, R. F. Von Cube, J. Von Den Driesch, C. Winter, R. Wolf, W. D. Zeuner, X. Zuo, G. Anagnostou, G. Daskalakis, A. Kyriakis, G. Melachroinos, Z. Painesis, I. Paraskevas, N. Saoulidou, K. Theofilatos, E. Tziaferi, E. Tzovara, K. Vellidis, I. Zisopoulos, T. Chatzistavrou, G. Karapostoli, K. Kousouris, E. Siamarkou, G. Tsipolitis, I. Bestintzanos, I. Evangelou, C. Foudas, P. Katsoulis, P. Kokkas, P. G. Kosmoglou Kioseoglou, N. Manthos, I. Papadopoulos, J. Strologas, C. Hajdu, D. Horvath, Á. Kadlecsik, C. Lee, K. Márton, A. J. Rádl, F. Sikler, V. Veszpremi, M. Csanád, K. Farkas, A. Fehérkuti, M. M. A. Gadallah, M. León Coello, G. Pásztor, G. I. Veres, B. Ujvari, G. Zilizi, G. Bencze, S. Czellar, J. Molnar, Z. Szillasi, T. Csorgo, F. Nemes, T. Novak, I. Szanyi, S. Bahinipati, S. Nayak, R. Raturi, S. Bansal, S. B. Beri, V. Bhatnagar, S. Chauhan, N. Dhingra, A. Kaur, H. Kaur, M. Kaur, S. Kumar, T. Sheokand, J. B. Singh, A. Singla, A. Bhardwaj, A. Chhetri, B. C. Choudhary, A. Kumar, A. Kumar, M. Naimuddin, S. Phor, K. Ranjan, M. K. Saini, P. Palni, S. Acharya, B. Gomber, S. Mukherjee, S. Bhattacharya, S. Das Gupta, S. Dutta, S. Dutta, S. Sarkar, M. M. Ameen, P. K. Behera, S. Chatterjee, G. Dash, A. Dattamunsi, P. Jana, P. Kalbhor, S. Kamble, J. R. Komaragiri, T. Mishra, P. R. Pujahari, A. K. Sikdar, R. K. Singh, P. Verma, S. Verma, A. Vijay, B. K. Sirasva, L. Bhatt, S. Dugad, G. B. Mohanty, M. Shelake, P. Suryadevara, A. Bala, S. Banerjee, S. Barman, R. M. Chatterjee, M. Guchait, S h. Jain, A. Jaiswal, S. Kumar, M. Maity, G. Majumder, K. Mazumdar, S. Parolia, R. Saxena, A. Thachayath, D. Maity, P. Mal, K. Naskar, A. Nayak, K. Pal, P. Sadangi, S. K. Swain, S. Varghese, D. Vats, S. Dube, P. Hazarika, B. Kansal, A. Laha, R. Sharma, S. Sharma, K. Y. Vaish, S. Ghosh, H. Bakhshiansohi, A. Jafari, V. Sedighzadeh Dalavi, M. Zeinali, S. Bashiri, S. Chenarani, S. M. Etesami, Y. Hosseini, M. Khakzad, E. Khazaie, M. Mohammadi Najafabadi, S. Tizchang, M. Felcini, M. Grunewald, M. Abbrescia, M. Barbieri, M. Buonsante, A. Colaleo, D. Creanza, N. De Filippis, M. De Palma, W. Elmetenawee, N. Ferrara, L. Fiore, L. Generoso, L. Longo, M. Louka, G. Maggi, M. Maggi, I. Margjeka, V. Mastrapasqua, S. My, F. Nenna, S. Nuzzo, A. Pellecchia, A. Pompili, G. Pugliese, R. Radogna, D. Ramos, A. Ranieri, L. Silvestris, F. M. Simone, Ü. Sözbilir, A. Stamerra, D. Troiano, R. Venditti, P. Verwilligen, A. Zaza, G. Abbiendi, C. Battilana, D. Bonacorsi, P. Capiluppi, F. R. Cavallo, M. Cuffiani, G. M. Dallavalle, T. Diotalevi, F. Fabbri, R. Farinelli, D. Fasanella, P. Giacomelli, C. Grandi, S. Lo Meo, M. Lorusso, L. Lunerti, S. Marcellini, G. Masetti, F. L. Navarria, G. Paggi, A. Perrotta, A. M. Rossi, S. Rossi Tisbeni, T. Rovelli, G. P. Siroli, S. Costa, A. Di Mattia, A. Lapertosa, R. Potenza, A. Tricomi, J. Altork, P. Assiouras, G. Barbagli, G. Bardelli, M. Bartolini, A. Calandri, B. Camaiani, A. Cassese, R. Ceccarelli, V. Ciulli, C. Civinini, R. D’Alessandro, L. Damenti, E. Focardi, T. Kello, G. Latino, P. Lenzi, M. Lizzo, M. Meschini, S. Paoletti, A. Papanastassiou, G. Sguazzoni, L. Viliani, L. Benussi, S. Bianco, S. Meola, D. Piccolo, M. Alves Gallo Pereira, F. Ferro, E. Robutti, S. Tosi, A. Benaglia, F. Brivio, V. Camagni, F. Cetorelli, F. De Guio, M. E. Dinardo, P. Dini, S. Gennai, R. Gerosa, A. Ghezzi, P. Govoni, L. Guzzi, M. R. Kim, G. Lavizzari, M. T. Lucchini, M. Malberti, S. Malvezzi, A. Massironi, D. Menasce, L. Moroni, M. Paganoni, S. Palluotto, D. Pedrini, A. Perego, G. Pizzati, T. Tabarelli de Fatis, S. Buontempo, C. Di Fraia, F. Fabozzi, L. Favilla, A. O. M. Iorio, L. Lista, P. Paolucci, B. Rossi, P. Azzi, N. Bacchetta, D. Bisello, L. Borella, P. Bortignon, G. Bortolato, A. C. M. Bulla, P. Checchia, T. Dorigo, F. Gasparini, U. Gasparini, S. Giorgetti, E. Lusiani, M. Margoni, G. Maron, A. T. Meneguzzo, J. Pazzini, F. Primavera, P. Ronchese, R. Rossin, F. Simonetto, M. Tosi, A. Triossi, S. Ventura, M. Zanetti, P. Zotto, A. Zucchetta, A. Braghieri, M. Brunoldi, S. Calzaferri, P. Montagna, M. Pelliccioni, V. Re, C. Riccardi, P. Salvini, I. Vai, P. Vitulo, S. Ajmal, M. E. Ascioti, G. M. Bilei, C. Carrivale, D. Ciangottini, L. Della Penna, L. Fanò, V. Mariani, M. Menichelli, F. Moscatelli, A. Rossi, A. Santocchia, D. Spiga, T. Tedeschi, C. Aimè, C. A. Alexe, P. Asenov, P. Azzurri, G. Bagliesi, L. Bianchini, T. Boccali, E. Bossini, D. Bruschini, R. Castaldi, F. Cattafesta, M. A. Ciocci, M. Cipriani, R. Dell’Orso, S. Donato, R. Forti, A. Giassi, F. Ligabue, A. C. Marini, A. Messineo, S. Mishra, V. K. Muraleedharan Nair Bindhu, S. Nandan, F. Palla, M. Riggirello, A. Rizzi, G. Rolandi, S. Roy Chowdhury, T. Sarkar, A. Scribano, P. Solanki, P. Spagnolo, F. Tenchini, R. Tenchini, G. Tonelli, N. Turini, F. Vaselli, A. Venturi, P. G. Verdini, P. Akrap, C. Basile, S. C. Behera, F. Cavallari, L. Cunqueiro Mendez, F. De Riggi, D. Del Re, M. Del Vecchio, E. Di Marco, M. Diemoz, F. Errico, L. Frosina, R. Gargiulo, B. Harikrishnan, F. Lombardi, E. Longo, L. Martikainen, J. Mijuskovic, G. Organtini, N. Palmeri, R. Paramatti, T. Pauletto, S. Rahatlou, C. Rovelli, F. Santanastasio, L. Soffi, V. Vladimirov, N. Amapane, R. Arcidiacono, S. Argiro, M. Arneodo, N. Bartosik, R. Bellan, A. Bellora, C. Biino, C. Borca, N. Cartiglia, M. Costa, R. Covarelli, N. Demaria, L. Finco, M. Grippo, B. Kiani, L. Lanteri, F. Legger, F. Luongo, C. Mariotti, S. Maselli, A. Mecca, L. Menzio, P. Meridiani, E. Migliore, M. Monteno, M. M. Obertino, G. Ortona, L. Pacher, N. Pastrone, M. Ruspa, F. Siviero, V. Sola, A. Solano, A. Staiano, C. Tarricone, D. Trocino, G. Umoret, E. Vlasov, R. White, J. Babbar, S. Belforte, V. Candelise, M. Casarsa, F. Cossutti, K. De Leo, G. Della Ricca, R. Delli Gatti, S. Dogra, J. Hong, J. Kim, T. Kim, D. Lee, H. Lee, J. Lee, S. W. Lee, C. S. Moon, Y. D. Oh, S. Sekmen, B. Tae, Y. C. Yang, M. S. Kim, G. Bak, P. Gwak, H. Kim, D. H. Moon, J. Seo, E. Asilar, F. Carnevali, J. Choi, T. J. Kim, Y. Ryou, J. Song, S. Ha, S. Han, B. Hong, J. Kim, K. Lee, K. S. Lee, S. Lee, J. Yoo, J. Goh, J. Shin, S. Yang, Y. Kang, H. S. Kim, Y. Kim, B. Ko, S. Lee, J. Almond, J. H. Bhyun, J. Choi, J. Choi, W. Jun, H. Kim, J. Kim, T. Kim, Y. Kim, Y. W. Kim, S. Ko, H. Lee, J. Lee, J. Lee, B. H. Oh, J. Shin, U. K. Yang, I. Yoon, W. Jang, D. Kim, S. Kim, J. S. H. Lee, Y. Lee, I. C. Park, Y. Roh, I. J. Watson, G. Cho, K. Hwang, B. Kim, S. Kim, K. Lee, H. D. Yoo, Y. Lee, I. Yu, T. Beyrouthy, Y. Gharbia, F. Alazemi, K. Dreimanis, O. M. Eberlins, A. Gaile, C. Munoz Diaz, D. Osite, G. Pikurs, R. Plese, A. Potrebko, M. Seidel, D. Sidiropoulos Kontos, N. R. Strautnieks, M. Ambrozas, A. Juodagalvis, S. Nargelas, A. Rinkevicius, G. Tamulaitis, I. Yusuff, Z. Zolkapli, J. F. Benitez, A. Castaneda Hernandez, A. Cota Rodriguez, L. E. Cuevas Picos, H. A. Encinas Acosta, L. G. Gallegos Maríñez, J. A. Murillo Quijada, L. Valencia Palomo, G. Ayala, H. Castilla-Valdez, H. Crotte Ledesma, R. Lopez-Fernandez, J. Mejia Guisao, R. Reyes-Almanza, A. Sánchez Hernández, C. Oropeza Barrera, D. L. Ramirez Guadarrama, M. Ramírez García, I. Bautista, F. E. Neri Huerta, I. Pedraza, H. A. Salazar Ibarguen, C. Uribe Estrada, I. Bubanja, N. Raicevic, P. H. Butler, A. Ahmad, M. I. Asghar, A. Awais, M. I. M. Awan, W. A. Khan, V. Avati, L. Forthomme, L. Grzanka, M. Malawski, K. Piotrzkowski, M. Bluj, M. Górski, M. Kazana, M. Szleper, P. Zalewski, K. Bunkowski, K. Doroba, A. Kalinowski, M. Konecki, J. Krolikowski, A. Muhammad, P. Fokow, K. Pozniak, W. Zabolotny, M. Araujo, D. Bastos, C. Beirão Da Cruz E Silva, A. Boletti, M. Bozzo, T. Camporesi, G. Da Molin, M. Gallinaro, J. Hollar, N. Leonardo, G. B. Marozzo, A. Petrilli, M. Pisano, J. Seixas, J. Varela, J. W. Wulff, P. Adzic, L. Markovic, P. Milenovic, V. Milosevic, D. Devetak, M. Dordevic, J. Milosevic, L. Nadderd, V. Rekovic, M. Stojanovic, M. Alcalde Martinez, J. Alcaraz Maestre, Cristina F. Bedoya, J. A. Brochero Cifuentes, Oliver M. Carretero, M. Cepeda, M. Cerrada, N. Colino, B. De La Cruz, A. Delgado Peris, A. Escalante Del Valle, D. Fernández Del Val, J. P. Fernández Ramos, J. Flix, M. C. Fouz, M. Gonzalez Hernandez, O. Gonzalez Lopez, S. Goy Lopez, J. M. Hernandez, M. I. Josa, J. Llorente Merino, C. Martin Perez, E. Martin Viscasillas, D. Moran, C. M. Morcillo Perez, Á. Navarro Tobar, R. Paz Herrera, A. Pérez-Calero Yzquierdo, J. Puerta Pelayo, I. Redondo, J. Vazquez Escobar, J. F. de Trocóniz, B. Alvarez Gonzalez, J. Ayllon Torresano, A. Cardini, J. Cuevas, J. Del Riego Badas, D. Estrada Acevedo, J. Fernandez Menendez, S. Folgueras, I. Gonzalez Caballero, P. Leguina, M. Obeso Menendez, E. Palencia Cortezon, J. Prado Pico, A. Soto Rodríguez, P. Vischia, S. Blanco Fernández, I. J. Cabrillo, A. Calderon, J. Duarte Campderros, M. Fernandez, G. Gomez, C. Lasaosa García, R. Lopez Ruiz, C. Martinez Rivero, P. Martinez Ruiz del Arbol, F. Matorras, P. Matorras Cuevas, E. Navarrete Ramos, J. Piedra Gomez, C. Quintana San Emeterio, L. Scodellaro, I. Vila, R. Vilar Cortabitarte, J. M. Vizan Garcia, B. Kailasapathy, D. D. C. Wickramarathna, W. G. D. Dharmaratna, K. Liyanage, N. Perera, D. Abbaneo, C. Amendola, R. Ardino, E. Auffray, J. Baechler, D. Barney, J. Bendavid, M. Bianco, A. Bocci, L. Borgonovi, C. Botta, A. Bragagnolo, C. E. Brown, C. Caillol, G. Cerminara, P. Connor, K. Cormier, D. d’Enterria, A. Dabrowski, A. David, A. De Roeck, M. M. Defranchis, M. Deile, M. Dobson, P. J. Fernández Manteca, B. A. Fontana Santos Alves, E. Fontanesi, W. Funk, A. Gaddi, S. Giani, D. Gigi, K. Gill, F. Glege, M. Glowacki, A. Gruber, J. Hegeman, J. K. Heikkilä, R. Hofsaess, B. Huber, T. James, P. Janot, O. Kaluzinska, O. Karacheban, G. Karathanasis, S. Laurila, P. Lecoq, E. Leutgeb, C. Lourenço, A.-M. Lyon, M. Magherini, L. Malgeri, M. Mannelli, A. Mehta, F. Meijers, J. A. Merlin, S. Mersi, E. Meschi, M. Migliorini, F. Monti, F. Moortgat, M. Mulders, M. Musich, I. Neutelings, S. Orfanelli, F. Pantaleo, M. Pari, G. Petrucciani, A. Pfeiffer, M. Pierini, M. Pitt, H. Qu, D. Rabady, A. Reimers, B. Ribeiro Lopes, F. Riti, P. Rosado, M. Rovere, H. Sakulin, R. Salvatico, S. Sanchez Cruz, S. Scarfi, M. Selvaggi, K. Shchelina, P. Silva, P. Sphicas, A. G. Stahl Leiton, A. Steen, S. Summers, D. Treille, P. Tropea, E. Vernazza, J. Wanczyk, S. Wuchterl, M. Zarucki, P. Zehetner, P. Zejdl, G. Zevi Della Porta, L. Caminada, W. Erdmann, R. Horisberger, Q. Ingram, H. C. Kaestli, D. Kotlinski, C. Lange, U. Langenegger, A. Nigamova, L. Noehte, T. Rohe, A. Samalan, T. K. Aarrestad, M. Backhaus, T. Bevilacqua, G. Bonomelli, C. Cazzaniga, K. Datta, P. De Bryas Dexmiers D’Archiacchiac, A. De Cosa, G. Dissertori, M. Dittmar, M. Donegà, F. Glessgen, C. Grab, N. Härringer, T. G. Harte, W. Lustermann, M. Malucchi, R. A. Manzoni, L. Marchese, A. Mascellani, F. Nessi-Tedaldi, F. Pauss, B. Ristic, R. Seidita, J. Steggemann, A. Tarabini, D. Valsecchi, R. Wallny, C. Amsler, P. Bärtschi, F. Bilandzija, M. F. Canelli, G. Celotto, V. Guglielmi, A. Jofrehei, B. Kilminster, T. H. Kwok, S. Leontsinis, V. Lukashenko, A. Macchiolo, F. Meng, M. Missiroli, J. Motta, P. Robmann, E. Shokr, F. Stäger, R. Tramontano, P. Viscone, D. Bhowmik, C. M. Kuo, P. K. Rout, S. Taj, P. C. Tiwari, L. Ceard, K. F. Chen, Z. g. Chen, A. De Iorio, W.-S. Hou, T. h. Hsu, Y. w. Kao, S. Karmakar, G. Kole, Y. y. Li, R.-S. Lu, E. Paganis, X. f. Su, J. Thomas-Wilsker, L. s. Tsai, D. Tsionou, H. y. Wu, E. Yazgan, C. Asawatangtrakuldee, N. Srimanobhas, Y. Maghrbi, D. Agyel, F. Dolek, I. Dumanoglu, Y. Guler, E. Gurpinar Guler, C. Isik, O. Kara, A. Kayis Topaksu, Y. Komurcu, G. Onengut, K. Ozdemir, B. Tali, U. G. Tok, E. Uslan, I. S. Zorbakir, S. Sen, M. Yalvac, B. Akgun, I. O. Atakisi, E. Gülmez, M. Kaya, O. Kaya, M. A. Sarkisla, S. Tekten, D. Boncukcu, A. Cakir, K. Cankocak, B. Hacisahinoglu, I. Hos, B. Kaynak, S. Ozkorucuklu, O. Potok, H. Sert, C. Simsek, C. Zorbilmez, S. Cerci, C. Dozen, B. Isildak, E. Simsek, D. Sunar Cerci, T. Yetkin, A. Boyaryntsev, O. Dadazhanova, B. Grynyov, L. Levchuk, J. J. Brooke, A. Bundock, F. Bury, E. Clement, D. Cussans, D. Dharmender, H. Flacher, J. Goldstein, H. F. Heath, M.-L. Holmberg, L. Kreczko, S. Paramesvaran, L. Robertshaw, M. S. Sanjrani, J. Segal, V. J. Smith, A. H. Ball, K. W. Bell, A. Belyaev, C. Brew, R. M. Brown, D. J. A. Cockerill, A. Elliot, K. V. Ellis, J. Gajownik, K. Harder, S. Harper, J. Linacre, K. Manolopoulos, M. Moallemi, D. M. Newbold, E. Olaiya, D. Petyt, T. Reis, A. R. Sahasransu, G. Salvi, T. Schuh, C. H. Shepherd-Themistocleous, I. R. Tomalin, K. C. Whalen, T. Williams, I. Andreou, R. Bainbridge, P. Bloch, O. Buchmuller, C. A. Carrillo Montoya, D. Colling, I. Das, P. Dauncey, G. Davies, M. Della Negra, S. Fayer, G. Fedi, G. Hall, H. R. Hoorani, A. Howard, G. Iles, C. R. Knight, P. Krueper, J. Langford, K. H. Law, J. León Holgado, L. Lyons, A.-M. Magnan, B. Maier, S. Mallios, A. Mastronikolis, M. Mieskolainen, J. Nash, M. Pesaresi, P. B. Pradeep, B. C. Radburn-Smith, A. Richards, A. Rose, L. Russell, K. Savva, R. Schmitz, C. Seez, R. Shukla, A. Tapper, K. Uchida, G. P. Uttley, T. Virdee, M. Vojinovic, N. Wardle, D. Winterbottom, J. E. Cole, A. Khan, P. Kyberd, I. D. Reid, S. Abdullin, A. Brinkerhoff, E. Collins, M. R. Darwish, J. Dittmann, K. Hatakeyama, V. Hegde, J. Hiltbrand, B. McMaster, J. Samudio, S. Sawant, C. Sutantawibul, J. Wilson, J. M. Hogan, R. Bartek, A. Dominguez, S. Raj, B. Sahu, A. E. Simsek, S. S. Yu, B. Bam, A. Buchot Perraguin, S. Campbell, R. Chudasama, S. I. Cooper, C. Crovella, G. Fidalgo, S. V. Gleyzer, A. Khukhunaishvili, K. Matchev, E. Pearson, P. Rumerio, E. Usai, R. Yi, S. Cholak, G. De Castro, Z. Demiragli, C. Erice, C. Fangmeier, C. Fernandez Madrazo, J. Fulcher, F. Golf, S. Jeon, J. O’Cain, I. Reed, J. Rohlf, K. Salyer, D. Sperka, D. Spitzbart, I. Suarez, A. Tsatsos, E. Wurtz, A. G. Zecchinelli, G. Barone, G. Benelli, D. Cutts, S. Ellis, L. Gouskos, M. Hadley, U. Heintz, K. W. Ho, T. Kwon, L. Lambrecht, G. Landsberg, K. T. Lau, J. Luo, S. Mondal, J. Roloff, T. Russell, S. Sagir, X. Shen, M. Stamenkovic, N. Venkatasubramanian, S. Abbott, S. Baradia, B. Barton, R. Breedon, H. Cai, M. Calderon De La Barca Sanchez, E. Cannaert, M. Chertok, M. Citron, J. Conway, P. T. Cox, F. Eble, R. Erbacher, O. Kukral, G. Mocellin, S. Ostrom, I. Salazar Segovia, J. S. Tafoya Vargas, W. Wei, S. Yoo, K. Adamidis, M. Bachtis, D. Campos, R. Cousins, S. Crossley, G. Flores Avila, J. Hauser, M. Ignatenko, M. A. Iqbal, T. Lam, Y. f. Lo, E. Manca, A. Nunez Del Prado, D. Saltzberg, V. Valuev, R. Clare, J. W. Gary, G. Hanson, A. Aportela, A. Arora, J. G. Branson, S. Cittolin, S. Cooperstein, B. D’Anzi, D. Diaz, J. Duarte, L. Giannini, Y. Gu, J. Guiang, V. Krutelyov, R. Lee, J. Letts, H. Li, M. Masciovecchio, F. Mokhtar, S. Mukherjee, M. Pieri, D. Primosch, M. Quinnan, V. Sharma, M. Tadel, E. Vourliotis, F. Würthwein, A. Yagil, Z. Zhao, A. Barzdukas, L. Brennan, C. Campagnari, S. Carron Montero, K. Downham, C. Grieco, M. M. Hussain, J. Incandela, M. W. K. Lai, A. J. Li, P. Masterson, J. Richman, S. N. Santpur, D. Stuart, T. Á. Vámi, X. Yan, D. Zhang, A. Albert, S. Bhattacharya, A. Bornheim, O. Cerri, R. Kansal, H. B. Newman, G. Reales Gutiérrez, T. Sievert, M. Spiropulu, J. R. Vlimant, R. A. Wynne, S. Xie, J. Alison, S. An, M. Cremonesi, V. Dutta, E. Y. Ertorer, T. Ferguson, T. A. Gómez Espinosa, A. Harilal, A. Kallil Tharayil, M. Kanemura, C. Liu, M. Marchegiani, P. Meiring, S. Murthy, P. Palit, K. Park, M. Paulini, A. Roberts, A. Sanchez, W. Terrill, J. P. Cumalat, W. T. Ford, A. Hart, S. Kwan, J. Pearkes, C. Savard, N. Schonbeck, K. Stenson, K. A. Ulmer, S. R. Wagner, N. Zipper, D. Zuolo, J. Alexander, X. Chen, J. Dickinson, A. Duquette, J. Fan, X. Fan, J. Grassi, S. Hogan, P. Kotamnives, J. Monroy, G. Niendorf, M. Oshiro, J. R. Patterson, A. Ryd, J. Thom, P. Wittich, R. Zou, L. Zygala, M. Albrow, M. Alyari, O. Amram, G. Apollinari, A. Apresyan, L. A. T. Bauerdick, D. Berry, J. Berryhill, P. C. Bhat, K. Burkett, J. N. Butler, A. Canepa, G. B. Cerati, H. W. K. Cheung, F. Chlebana, C. Cosby, G. Cummings, I. Dutta, V. D. Elvira, J. Freeman, A. Gandrakota, Z. Gecse, L. Gray, D. Green, A. Grummer, S. Grünendahl, D. Guerrero, O. Gutsche, R. M. Harris, J. Hirschauer, V. Innocente, B. Jayatilaka, S. Jindariani, M. Johnson, U. Joshi, R. S. Kim, B. Klima, S. Lammel, D. Lincoln, R. Lipton, T. Liu, K. Maeshima, D. Mason, P. McBride, P. Merkel, S. Mrenna, S. Nahn, J. Ngadiuba, D. Noonan, S. Norberg, V. Papadimitriou, N. Pastika, K. Pedro, C. Pena, C. E. Perez Lara, V. Perovic, F. Ravera, A. Reinsvold Hall, L. Ristori, M. Safdari, E. Sexton-Kennedy, E. Smith, N. Smith, A. Soha, L. Spiegel, S. Stoynev, J. Strait, L. Taylor, S. Tkaczyk, N. V. Tran, L. Uplegger, E. W. Vaandering, C. Wang, I. Zoi, C. Aruta, P. Avery, D. Bourilkov, P. Chang, V. Cherepanov, R. D. Field, C. Huh, E. Koenig, M. Kolosova, J. Konigsberg, A. Korytov, G. Mitselmakher, K. Mohrman, A. Muthirakalayil Madhu, N. Rawal, S. Rosenzweig, V. Sulimov, Y. Takahashi, J. Wang, T. Adams, A. Al Kadhim, A. Askew, S. Bower, R. Goff, R. Hashmi, A. Hassani, T. Kolberg, G. Martinez, M. Mazza, H. Prosper, P. R. Prova, R. Yohay, B. Alsufyani, S. Butalla, S. Das, M. Hohlmann, M. Lavinsky, E. Yanes, M. R. Adams, N. Barnett, A. Baty, C. Bennett, R. Cavanaugh, R. Escobar Franco, O. Evdokimov, C. E. Gerber, H. Gupta, M. Hawksworth, A. Hingrajiya, D. J. Hofman, Z. Huang, J. h. Lee, C. Mills, S. Nanda, G. Nigmatkulov, B. Ozek, T. Phan, D. Pilipovic, R. Pradhan, E. Prifti, P. Roy, T. Roy, D. Shekar, N. Singh, A. Thielen, M. B. Tonjes, N. Varelas, M. A. Wadud, J. Yoo, M. Alhusseini, D. Blend, K. Dilsiz, O. K. Köseyan, A. Mestvirishvili, O. Neogi, H. Ogul, Y. Onel, A. Penzo, C. Snyder, E. Tiras, B. Blumenfeld, J. Davis, A. V. Gritsan, L. Kang, S. Kyriacou, P. Maksimovic, M. Roguljic, S. Sekhar, M. V. Srivastav, M. Swartz, A. Abreu, L. F. Alcerro Alcerro, J. Anguiano, S. Arteaga Escatel, P. Baringer, A. Bean, R. Bhattacharya, Z. Flowers, D. Grove, J. King, G. Krintiras, M. Lazarovits, C. Le Mahieu, J. Marquez, M. Murray, M. Nickel, S. Popescu, C. Rogan, C. Royon, S. Rudrabhatla, S. Sanders, C. Smith, G. Wilson, B. Allmond, N. Islam, A. Ivanov, K. Kaadze, Y. Maravin, J. Natoli, G. G. Reddy, D. Roy, G. Sorrentino, A. Baden, A. Belloni, J. Bistany-riebman, S. C. Eno, N. J. Hadley, S. Jabeen, R. G. Kellogg, T. Koeth, B. Kronheim, S. Lascio, P. Major, A. C. Mignerey, C. Palmer, C. Papageorgakis, M. M. Paranjpe, E. Popova, A. Shevelev, L. Zhang, C. Baldenegro Barrera, H. Bossi, S. Bright-Thonney, I. A. Cali, Y. c. Chen, P. c. Chou, M. D’Alfonso, J. Eysermans, C. Freer, G. Gomez-Ceballos, M. Goncharov, G. Grosso, P. Harris, D. Hoang, G. M. Innocenti, K. Ivanov, G. Kopp, D. Kovalskyi, J. Krupa, L. Lavezzo, Y.-J. Lee, K. Long, C. Mcginn, A. Novak, M. I. Park, C. Paus, C. Reissel, C. Roland, G. Roland, S. Rothman, T. a. Sheng, G. S. F. Stephans, D. Walter, J. Wang, Z. Wang, B. Wyslouch, T. J. Yang, A. Alpana, B. Crossman, W. J. Jackson, C. Kapsiak, M. Krohn, D. Mahon, J. Mans, B. Marzocchi, R. Rusack, O. Sancar, R. Saradhy, N. Strobbe, K. Bloom, D. R. Claes, G. Haza, J. Hossain, C. Joo, I. Kravchenko, K. H. M. Kwok, A. Rohilla, J. E. Siado, W. Tabb, A. Vagnerini, A. Wightman, F. Yan, H. Bandyopadhyay, L. Hay, H. w. Hsia, I. Iashvili, A. Kalogeropoulos, A. Kharchilava, A. Mandal, M. Morris, D. Nguyen, S. Rappoccio, H. Rejeb Sfar, A. Williams, D. Yu, A. Aarif, G. Alverson, E. Barberis, J. Bonilla, B. Bylsma, M. Campana, J. Dervan, Y. Haddad, Y. Han, I. Israr, A. Krishna, M. Lu, N. Manganelli, R. Mccarthy, D. M. Morse, T. Orimoto, L. Skinnari, C. S. Thoreson, E. Tsai, D. Wood, S. Dittmer, K. A. Hahn, M. Mcginnis, Y. Miao, D. G. Monk, M. H. Schmitt, A. Taliercio, M. Velasco, J. Wang, G. Agarwal, R. Band, R. Bucci, S. Castells, A. Das, A. Datta, A. Ehnis, R. Goldouzian, M. Hildreth, K. Hurtado Anampa, T. Ivanov, C. Jessop, A. Karneyeu, K. Lannon, J. Lawrence, N. Loukas, L. Lutton, J. Mariano, N. Marinelli, T. McCauley, C. Mcgrady, C. Moore, Y. Musienko, H. Nelson, M. Osherson, A. Piccinelli, R. Ruchti, A. Townsend, Y. Wan, M. Wayne, H. Yockey, M. Carrigan, R. De Los Santos, L. S. Durkin, C. Hill, M. Joyce, D. A. Wenzl, B. L. Winer, B. R. Yates, H. Bouchamaoui, G. Dezoort, P. Elmer, A. Frankenthal, M. Galli, B. Greenberg, N. Haubrich, K. Kennedy, Y. Lai, D. Lange, A. Loeliger, D. Marlow, I. Ojalvo, J. Olsen, F. Simpson, D. Stickland, C. Tully, S. Malik, R. Sharma, S. Chandra, A. Gu, L. Gutay, M. Huwiler, M. Jones, A. W. Jung, D. Kondratyev, J. Li, M. Liu, G. Negro, N. Neumeister, G. Paspalaki, S. Piperov, N. R. Saha, J. F. Schulte, F. Wang, A. Wildridge, W. Xie, Y. Yao, Y. Zhong, N. Parashar, A. Pathak, E. Shumka, D. Acosta, A. Agrawal, C. Arbour, T. Carnahan, P. Das, K. M. Ecklund, F. J. M. Geurts, T. Huang, I. Krommydas, N. Lewis, W. Li, J. Lin, O. Miguel Colin, B. P. Padley, R. Redjimi, J. Rotter, C. Vico Villalba, M. Wulansatiti, E. Yigitbasi, Y. Zhang, O. Bessidskaia Bylund, A. Bodek, P. de Barbaro, R. Demina, A. Garcia-Bellido, H. S. Hare, O. Hindrichs, N. Parmar, P. Parygin, H. Seo, R. Taus, Y. h. Yu, B. Chiarito, J. P. Chou, S. V. Clark, S. Donnelly, D. Gadkari, Y. Gershtein, E. Halkiadakis, C. Houghton, D. Jaroslawski, A. Kobert, I. Laflotte, A. Lath, J. Martins, M. Perez Prada, B. Rand, J. Reichert, P. Saha, S. Salur, S. Somalwar, R. Stone, S. A. Thayil, S. Thomas, J. Vora, D. Ally, A. G. Delannoy, S. Fiorendi, J. Harris, T. Holmes, A. R. Kanuganti, N. Karunarathna, J. Lawless, L. Lee, E. Nibigira, B. Skipworth, S. Spanier, D. Aebi, M. Ahmad, T. Akhter, K. Androsov, A. Basnet, A. Bolshov, O. Bouhali, A. Cagnotta, V. D’Amante, R. Eusebi, P. Flanagan, J. Gilmore, Y. Guo, T. Kamon, S. Luo, R. Mueller, A. Safonov, N. Akchurin, J. Damgov, Y. Feng, N. Gogate, W. Jin, S. W. Lee, C. Madrid, A. Mankel, T. Peltola, I. Volobouev, E. Appelt, Y. Chen, S. Greene, A. Gurrola, W. Johns, R. Kunnawalkam Elayavalli, A. Melo, D. Rathjens, F. Romeo, P. Sheldon, S. Tuo, J. Velkovska, J. Viinikainen, J. Zhang, B. Cardwell, H. Chung, B. Cox, J. Hakala, G. Hamilton Ilha Machado, R. Hirosky, M. Jose, A. Ledovskoy, C. Mantilla, C. Neu, C. Ramón Álvarez, Z. Wu, S. Bhattacharya, P. E. Karchin, A. Aravind, S. Banerjee, K. Black, T. Bose, E. Chavez, S. Dasu, P. Everaerts, C. Galloni, H. He, M. Herndon, A. Herve, C. K. Koraka, S. Lomte, R. Loveless, A. Mallampalli, A. Mohammadi, S. Mondal, T. Nelson, G. Parida, L. Pétré, D. Pinna, A. Savin, V. Shang, V. Sharma, W. H. Smith, D. Teague, A. Warden, S. Afanasiev, V. Alexakhin, Y u. Andreev, T. Aushev, D. Budkouski, R. Chistov, M. Danilov, T. Dimova, A. Ershov, S. Gninenko, I. Gorbunov, A. Kamenev, V. Karjavine, M. Kirsanov, V. Klyukhin, O. Kodolova, V. Korenkov, I. Korsakov, A. Kozyrev, N. Krasnikov, A. Lanev, A. Malakhov, V. Matveev, A. Nikitenko, V. Palichik, V. Perelygin, S. Petrushanko, O. Radchenko, M. Savina, V. Shalaev, S. Shmatov, S. Shulha, Y. Skovpen, K. Slizhevskiy, V. Smirnov, O. Teryaev, I. Tlisova, A. Toropin, N. Voytishin, A. Zarubin, I. Zhizhin, E. Boos, V. Bunichev, M. Dubinin, A. Gribushin, V. Savrin, A. Snigirev, L. Dudko, V. Kim, V. Murzin, V. Oreshkin, D. Sosnov

**Affiliations:** 1 Yerevan Physics Institute, Yerevan, Armenia; 2 Institut für Hochenergiephysik, Vienna, Austria; 3 Universiteit Antwerpen, Antwerpen, Belgium; 4 Vrije Universiteit Brussel, Brussel, Belgium; 5 Université Libre de Bruxelles, Bruxelles, Belgium; 6 Ghent University, Ghent, Belgium; 7 Université Catholique de Louvain, Louvain-la-Neuve, Belgium; 8 Centro Brasileiro de Pesquisas Fisicas, Rio de Janeiro, Brazil; 9 Universidade do Estado do Rio de Janeiro, Rio de Janeiro, Brazil; 10 Universidade Estadual Paulista, Universidade Federal do ABC, São Paulo, Brazil; 11 Institute for Nuclear Research and Nuclear Energy, Bulgarian Academy of Sciences, Sofia, Bulgaria; 12 University of Sofia, Sofia, Bulgaria; 13 Instituto De Alta Investigación, Universidad de Tarapacá, Casilla 7 D, Arica, Chile; 14 Universidad Tecnica Federico Santa Maria, Valparaiso, Chile; 15 Beihang University, Beijing, China; 16 Department of Physics, Tsinghua University, Beijing, China; 17 Institute of High Energy Physics, Beijing, China; 18 State Key Laboratory of Nuclear Physics and Technology, Peking University, Beijing, China; 19 State Key Laboratory of Nuclear Physics and Technology, Institute of Quantum Matter, South China Normal University, Guangzhou, China; 20 Sun Yat-Sen University, Guangzhou, China; 21 University of Science and Technology of China, Hefei, China; 22 Nanjing Normal University, Nanjing, China; 23 Institute of Modern Physics and Key Laboratory of Nuclear Physics and Ion-beam Application (MOE)-Fudan University, Shanghai, China; 24 Zhejiang University, Hangzhou, Zhejiang, China; 25 Universidad de Los Andes, Bogota, Colombia; 26 Universidad de Antioquia, Medellin, Colombia; 27 University of Split, Faculty of Electrical Engineering, Mechanical Engineering and Naval Architecture, Split, Croatia; 28 University of Split, Faculty of Science, Split, Croatia; 29 Institute Rudjer Boskovic, Zagreb, Croatia; 30 University of Cyprus, Nicosia, Cyprus; 31 Charles University, Prague, Czech Republic; 32 Escuela Politecnica Nacional, Quito, Ecuador; 33 Universidad San Francisco de Quito, Quito, Ecuador; 34 Academy of Scientific Research and Technology of the Arab Republic of Egypt, Egyptian Network of High Energy Physics, Cairo, Egypt; 35 Center for High Energy Physics (CHEP-FU), Fayoum University, El-Fayoum, Egypt; 36 National Institute of Chemical Physics and Biophysics, Tallinn, Estonia; 37 Department of Physics, University of Helsinki, Helsinki, Finland; 38 Helsinki Institute of Physics, Helsinki, Finland; 39 Lappeenranta-Lahti University of Technology, Lappeenranta, Finland; 40 IRFU, CEA, Université Paris-Saclay, Gif-sur-Yvette, France; 41 Laboratoire Leprince-Ringuet, CNRS/IN2P3, Ecole Polytechnique, Institut Polytechnique de Paris, Palaiseau, France; 42 Université de Strasbourg, CNRS, IPHC UMR 7178, Strasbourg, France; 43 Centre de Calcul de l’Institut National de Physique Nucleaire et de Physique des Particules, CNRS/IN2P3, Villeurbanne, France; 44 Institut de Physique des 2 Infinis de Lyon (IP2I ), Villeurbanne, France; 45 Georgian Technical University, Tbilisi, Georgia; 46 RWTH Aachen University, I. Physikalisches Institut, Aachen, Germany; 47 RWTH Aachen University, III. Physikalisches Institut A, Aachen, Germany; 48 RWTH Aachen University, III. Physikalisches Institut B, Aachen, Germany; 49 Deutsches Elektronen-Synchrotron, Hamburg, Germany; 50 University of Hamburg, Hamburg, Germany; 51 Karlsruher Institut fuer Technologie, Karlsruhe, Germany; 52 Institute of Nuclear and Particle Physics (INPP), NCSR Demokritos, Aghia Paraskevi, Greece; 53 National and Kapodistrian University of Athens, Athens, Greece; 54 National Technical University of Athens, Athens, Greece; 55 University of Ioánnina, Ioánnina, Greece; 56 HUN-REN Wigner Research Centre for Physics, Budapest, Hungary; 57 MTA-ELTE Lendület CMS Particle and Nuclear Physics Group, Eötvös Loránd University, Budapest, Hungary; 58 Faculty of Informatics, University of Debrecen, Debrecen, Hungary; 59 HUN-REN ATOMKI - Institute of Nuclear Research, Debrecen, Hungary; 60 Karoly Robert Campus, MATE Institute of Technology, Gyongyos, Hungary; 61 IIT Bhubaneswar, Bhubaneswar, India; 62 Panjab University, Chandigarh, India; 63 University of Delhi, Delhi, India; 64 Indian Institute of Technology Mandi (IIT-Mandi), Himachal Pradesh, India; 65 University of Hyderabad, Hyderabad, India; 66 Indian Institute of Technology Kanpur, Kanpur, India; 67 Saha Institute of Nuclear Physics, HBNI, Kolkata, India; 68 Indian Institute of Technology Madras, Madras, India; 69 IISER Mohali, India, Mohali, India; 70 Tata Institute of Fundamental Research-A, Mumbai, India; 71 Tata Institute of Fundamental Research-B, Mumbai, India; 72 National Institute of Science Education and Research, An OCC of Homi Bhabha National Institute, Bhubaneswar, Odisha India; 73 Indian Institute of Science Education and Research (IISER), Pune, India; 74 Indian Institute of Technology Hyderabad, Telangana, India; 75 Isfahan University of Technology, Isfahan, Iran; 76 Institute for Research in Fundamental Sciences (IPM), Tehran, Iran; 77 University College Dublin, Dublin, Ireland; 78 INFN Sezione di Bari, Università di Bari, Politecnico di Bari, Bari, Italy; 79 INFN Sezione di Bologna, Università di Bologna, Bologna, Italy; 80 INFN Sezione di Catania, Università di Catania, Catania, Italy; 81 INFN Sezione di Firenze, Università di Firenze, Firenze, Italy; 82 INFN Laboratori Nazionali di Frascati, Frascati, Italy; 83 INFN Sezione di Genova, Università di Genova, Genoa, Italy; 84 INFN Sezione di Milano-Bicocca, Università di Milano-Bicocca, Milan, Italy; 85 INFN Sezione di Napoli, Università di Napoli ’Federico II’, Napoli, Italy; Università della Basilicata, Potenza, Italy; Scuola Superiore Meridionale (SSM), Naples, Italy; 86 INFN Sezione di Padova, Università di Padova, Padova, Italy; Universita degli Studi di Cagliari, Cagliari, Italy; 87 INFN Sezione di Pavia, Università di Pavia, Pavia, Italy; 88 INFN Sezione di Perugia, Università di Perugia, Perugia, Italy; 89 INFN Sezione di Pisa, Università di Pisa, Scuola Normale Superiore di Pisa, Pisa, Italy; Università di Siena, Siena, Italy; 90 INFN Sezione di Roma, Sapienza Università di Roma, Rome, Italy; 91 INFN Sezione di Torino, Università di Torino, Torino, Italy; Università del Piemonte Orientale, Novara, Italy; 92 INFN Sezione di Trieste, Università di Trieste, Trieste, Italy; 93 Kyungpook National University, Daegu, Korea; 94 Department of Mathematics and Physics - GWNU, Gangneung, Korea; 95 Chonnam National University, Institute for Universe and Elementary Particles, Kwangju, Korea; 96 Hanyang University, Seoul, Korea; 97 Korea University, Seoul, Korea; 98 Department of Physics, Kyung Hee University, Seoul, Korea; 99 Sejong University, Seoul, Korea; 100 Seoul National University, Seoul, Korea; 101 University of Seoul, Seoul, Korea; 102 Department of Physics, Yonsei University, Seoul, Korea; 103 Sungkyunkwan University, Suwon, Korea; 104 College of Engineering and Technology, American University of the Middle East (AUM), Dasman, Kuwait; 105 Kuwait University-College of Science-Department of Physics, Safat, Kuwait; 106 Riga Technical University, Riga, Latvia; 107 University of Latvia (LU), Riga, Latvia; 108 Vilnius University, Vilnius, Lithuania; 109 National Centre for Particle Physics, Universiti Malaya, Kuala Lumpur, Malaysia; 110 Universidad de Sonora (UNISON), Hermosillo, Mexico; 111 Centro de Investigacion y de Estudios Avanzados del IPN, Mexico City, Mexico; 112 Universidad Iberoamericana, Mexico City, Mexico; 113 Benemerita Universidad Autonoma de Puebla, Puebla, Mexico; 114 University of Montenegro, Podgorica, Montenegro; 115 University of Canterbury, Christchurch, New Zealand; 116 National Centre for Physics, Quaid-I-Azam University, Islamabad, Pakistan; 117 AGH University of Krakow, Krakow, Poland; 118 National Centre for Nuclear Research, Swierk, Poland; 119 Institute of Experimental Physics, Faculty of Physics, University of Warsaw, Warsaw, Poland; 120 Warsaw University of Technology, Warsaw, Poland; 121 Laboratório de Instrumentação e Física Experimental de Partículas, Lisbon, Portugal; 122 Faculty of Physics, University of Belgrade, Belgrade, Serbia; 123 VINCA Institute of Nuclear Sciences, University of Belgrade, Belgrade, Serbia; 124 Centro de Investigaciones Energéticas Medioambientales y Tecnológicas (CIEMAT), Madrid, Spain; 125 Universidad Autónoma de Madrid, Madrid, Spain; 126 Universidad de Oviedo, Instituto Universitario de Ciencias y Tecnologías Espaciales de Asturias (ICTEA), Oviedo, Spain; 127 Instituto de Física de Cantabria (IFCA), CSIC-Universidad de Cantabria, Santander, Spain; 128 University of Colombo, Colombo, Sri Lanka; 129 University of Ruhuna, Department of Physics, Matara, Sri Lanka; 130 CERN, European Organization for Nuclear Research, Geneva, Switzerland; 131 PSI Center for Neutron and Muon Sciences, Villigen, Switzerland; 132 ETH Zurich-Institute for Particle Physics and Astrophysics (IPA), Zurich, Switzerland; 133 Universität Zürich, Zurich, Switzerland; 134 National Central University, Chung-Li, Taiwan; 135 National Taiwan University (NTU), Taipei, Taiwan; 136 High Energy Physics Research Unit, Department of Physics, Faculty of Science, Chulalongkorn University, Bangkok, Thailand; 137 Tunis El Manar University, Tunis, Tunisia; 138 Çukurova University, Physics Department, Science and Art Faculty, Adana, Turkey; 139 Hacettepe University, Ankara, Turkey; 140 Physics Department, Middle East Technical University, Ankara, Turkey; 141 Bogazici University, Istanbul, Turkey; 142 Istanbul Technical University, Istanbul, Turkey; 143 Istanbul University, Istanbul, Turkey; 144 Yildiz Technical University, Istanbul, Turkey; 145 Institute for Scintillation Materials of National Academy of Science of Ukraine, Kharkiv, Ukraine; 146 National Science Centre, Kharkiv Institute of Physics and Technology, Kharkiv, Ukraine; 147 University of Bristol, Bristol, UK; 148 Rutherford Appleton Laboratory, Didcot, UK; 149 Imperial College, London, UK; 150 Brunel University, Uxbridge, UK; 151 Baylor University, Waco, TX USA; 152 Bethel University, St. Paul, MN USA; 153 Catholic University of America, Washington, DC USA; 154 The University of Alabama, Tuscaloosa, AL USA; 155 Boston University, Boston, MA USA; 156 Brown University, Providence, RI USA; 157 University of California, Davis, Davis, CA USA; 158 University of California, Los Angeles, CA USA; 159 University of California, Riverside, Riverside, CA USA; 160 University of California, San Diego, La Jolla, CA USA; 161 Department of Physics, University of California, Santa Barbara, Santa Barbara, CA USA; 162 California Institute of Technology, Pasadena, CA USA; 163 Carnegie Mellon University, Pittsburgh, PA USA; 164 University of Colorado Boulder, Boulder, CO USA; 165 Cornell University, Ithaca, NY USA; 166 Fermi National Accelerator Laboratory, Batavia, IL USA; 167 University of Florida, Gainesville, FL USA; 168 Florida State University, Tallahassee, FL USA; 169 Florida Institute of Technology, Melbourne, FL USA; 170 University of Illinois Chicago, Chicago, IL USA; 171 The University of Iowa, Iowa City, IA USA; 172 Johns Hopkins University, Baltimore, MD USA; 173 The University of Kansas, Lawrence, KS USA; 174 Kansas State University, Manhattan, KS USA; 175 University of Maryland, College Park, MD USA; 176 Massachusetts Institute of Technology, Cambridge, MA USA; 177 University of Minnesota, Minneapolis, MN USA; 178 University of Nebraska-Lincoln, Lincoln, NE USA; 179 State University of New York at Buffalo, Buffalo, NY USA; 180 Northeastern University, Boston, MA USA; 181 Northwestern University, Evanston, IL USA; 182 University of Notre Dame, Notre Dame, IN USA; 183 The Ohio State University, Columbus, OH USA; 184 Princeton University, Princeton, NJ USA; 185 University of Puerto Rico, Mayaguez, PR USA; 186 Purdue University, West Lafayette, IN USA; 187 Purdue University Northwest, Hammond, IN USA; 188 Rice University, Houston, TX USA; 189 University of Rochester, Rochester, NY USA; 190 Rutgers, The State University of New Jersey, Piscataway, NJ USA; 191 University of Tennessee, Knoxville, TN USA; 192 Texas A&M University, College Station, TX USA; 193 Texas Tech University, Lubbock, TX USA; 194 Vanderbilt University, Nashville, TN USA; 195 University of Virginia, Charlottesville, VA USA; 196 Wayne State University, Detroit, MI USA; 197 University of Wisconsin-Madison, Madison, WI USA; 198 Authors affiliated with an international laboratory covered by a cooperation agreement with CERN, Geneva, Switzerland; 199 Authors affiliated with an institute formerly covered by a cooperation agreement with CERN, Geneva, Switzerland; 200 Yerevan State University, Yerevan, Armenia; 201 TU Wien, Vienna, Austria; 202 Ghent University, Ghent, Belgium; 203 FACAMP - Faculdades de Campinas, Sao Paulo, Brazil; 204 Universidade Estadual de Campinas, Campinas, Brazil; 205 Federal University of Rio Grande do Sul, Porto Alegre, Brazil; 206 The University of the State of Amazonas, Manaus, Brazil; 207 University of Chinese Academy of Sciences, Beijing, China; 208 University of Chinese Academy of Sciences, Beijing, China; 209 School of Physics, Zhengzhou University, Zhengzhou, China; 210 Now at Henan Normal University, Xinxiang, China; 211 University of Shanghai for Science and Technology, Shanghai, China; 212 The University of Iowa, Iowa City, Iowa, USA; 213 Nanjing Normal University, Nanjing, China; 214 Center for High Energy Physics, Peking University, Beijing, China; 215 Helwan University, Cairo, Egypt; 216 Now at Zewail City of Science and Technology, Zewail, Egypt; 217 Cairo University, Cairo, Egypt; 218 Université de Haute Alsace, Mulhouse, France; 219 Purdue University, West Lafayette, Indiana, USA; 220 an Institute Formerly Covered by a Cooperation Agreement with CERN, Geneva, Switzerland; 221 University of Hamburg, Hamburg, Germany; 222 RWTH Aachen University, III. Physikalisches Institut A, Aachen, Germany; 223 Bergische University Wuppertal (BUW), Wuppertal, Germany; 224 Brandenburg University of Technology, Cottbus, Germany; 225 Forschungszentrum Jülich, Juelich, Germany; 226 CERN, European Organization for Nuclear Research, Geneva, Switzerland; 227 HUN-REN ATOMKI - Institute of Nuclear Research, Debrecen, Hungary; 228 Now at Universitatea Babes-Bolyai - Facultatea de Fizica, Cluj-Napoca, Romania; 229 MTA-ELTE Lendület CMS Particle and Nuclear Physics Group, Eötvös Loránd University, Budapest, Hungary; 230 HUN-REN Wigner Research Centre for Physics, Budapest, Hungary; 231 Physics Department, Faculty of Science, Assiut University, Assiut, Egypt; 232 The University of Kansas, Lawrence, Kansas, USA; 233 Punjab Agricultural University, Ludhiana, India; 234 University of Hyderabad, Hyderabad, India; 235 Indian Institute of Science (IISc), Bangalore, India; 236 University of Visva-Bharati, Santiniketan, India; 237 Institute of Physics, Bhubaneswar, India; 238 Deutsches Elektronen-Synchrotron, Hamburg, Germany; 239 Isfahan University of Technology, Isfahan, Iran; 240 Sharif University of Technology, Tehran, Iran; 241 Department of Physics, University of Science and Technology of Mazandaran, Behshahr, Iran; 242 Department of Physics, Faculty of Science, Arak University, ARAK, Iran; 243 Italian National Agency for New Technologies, Energy and Sustainable Economic Development, Bologna, Italy; 244 Centro Siciliano di Fisica Nucleare e di Struttura Della Materia, Catania, Italy; 245 Università degli Studi Guglielmo Marconi, Roma, Italy; 246 Scuola Superiore Meridionale, Università di Napoli ’Federico II’, Napoli, Italy; 247 Fermi National Accelerator Laboratory, Batavia, Illinois, USA; 248 Lulea University of Technology, Lulea, Sweden; 249 Laboratori Nazionali di Legnaro dell’INFN, Legnaro, Italy; 250 Consiglio Nazionale delle Ricerche - Istituto Officina dei Materiali, Perugia, Italy; 251 UPES - University of Petroleum and Energy Studies, Dehradun, India; 252 Institut de Physique des 2 Infinis de Lyon (IP2I ), Villeurbanne, France; 253 Department of Applied Physics, Faculty of Science and Technology, Universiti Kebangsaan Malaysia, Bangi, Malaysia; 254 Trincomalee Campus, Eastern University, Sri Lanka, Nilaveli, Sri Lanka; 255 Saegis Campus, Nugegoda, Sri Lanka; 256 National and Kapodistrian University of Athens, Athens, Greece; 257 Ecole Polytechnique Fédérale Lausanne, Lausanne, Switzerland; 258 Universität Zürich, Zurich, Switzerland; 259 Stefan Meyer Institute for Subatomic Physics, Vienna, Austria; 260 Near East University, Research Center of Experimental Health Science, Mersin, Turkey; 261 Konya Technical University, Konya, Turkey; 262 Istanbul Topkapi University, Istanbul, Turkey; 263 Izmir Bakircay University, Izmir, Turkey; 264 Adiyaman University, Adiyaman, Turkey; 265 Bozok Universitetesi Rektörlügü, Yozgat, Turkey; 266 Istanbul Sabahattin Zaim University, Istanbul, Turkey; 267 Marmara University, Istanbul, Turkey; 268 Milli Savunma University, Istanbul, Turkey; 269 Informatics and Information Security Research Center, Gebze/Kocaeli, Turkey; 270 Kafkas University, Kars, Turkey; 271 Now at Istanbul Okan University, Istanbul, Turkey; 272 Istanbul University - Cerrahpasa, Faculty of Engineering, Istanbul, Turkey; 273 Istinye University, Istanbul, Turkey; 274 School of Physics and Astronomy, University of Southampton, Southampton, United Kingdom; 275 Monash University, Faculty of Science, Clayton, Australia; 276 Università di Torino, Torino, Italy; 277 Karamanoğlu Mehmetbey University, Karaman, Turkey; 278 California Lutheran University;, Thousand Oaks, California, USA; 279 California Institute of Technology, Pasadena, California, USA; 280 United States Naval Academy, Annapolis, Maryland, USA; 281 Bingol University, Bingol, Turkey; 282 Georgian Technical University, Tbilisi, Georgia; 283 Sinop University, Sinop, Turkey; 284 Erciyes University, Kayseri, Turkey; 285 Horia Hulubei National Institute of Physics and Nuclear Engineering (IFIN-HH), Bucharest, Romania; 286 Now at Another Institute Formerly Covered by a Cooperation Agreement with CERN, Geneva, Switzerland; 287 Hamad Bin Khalifa University (HBKU), Doha, Qatar; 288 Yerevan Physics Institute, Yerevan, Armenia; 289 Imperial College, London, United Kingdom; 290 CERN, 1211 Geneva 23, Switzerland

## Abstract

A search is presented for massive narrow-width resonances in the mass range of 1–4.5$\,\text {Te}\hspace{-.08em}\text {V}$, decaying into pairs of Higgs bosons (HH). The search uses proton–proton collision data at a center-of-mass energy of 13$\,\text {Te}\hspace{-.08em}\text {V}$   collected with the CMS detector at the CERN LHC during 2016–2018, corresponding to an integrated luminosity of 138$\,\text {fb}^{-1}$. The analysis targets final states where one Higgs boson decays into a pair of bottom quarks and the other into a pair of tau leptons, ${\text {X}} \rightarrow {\text {HH}} \rightarrow \text {b}{\bar{\text {b}}}\,\tau ^{+}\tau ^{-}$. It uses a single large radius jet to reconstruct the ${\text {H}} \rightarrow \text {b}{\bar{\text {b}}}$ decay, while the ${\text {H}} \rightarrow \tau ^{+}\tau ^{-}$ decay products can either be contained within a single large radius jet or appear as two isolated tau leptons. The observed data are consistent with standard model background expectations. Upper limits at 95% confidence level are set on the production cross section for resonant HH production for masses between 1 and 4.5$\,\text {Te}\hspace{-.08em}\text {V}$. This analysis sets the most sensitive limits to date on ${\text {X}} \rightarrow {\text {HH}} \rightarrow \text {b}\bar{\hbox {b}}\, \tau ^{+}\tau ^{-}$ decays in the mass range of 1.4–4.5$\,\text {Te}\hspace{-.08em}\text {V}$

## Introduction

The discovery of the Higgs boson (H) by the CMS [1, 2] and ATLAS [3, 4] experiments confirmed the standard model (SM) as a robust theoretical framework for describing high-energy interactions. However, unresolved questions within the SM, such as the hierarchy problem [5], have driven the search for beyond-the-SM (BSM) physics.

**Fig. 1:**
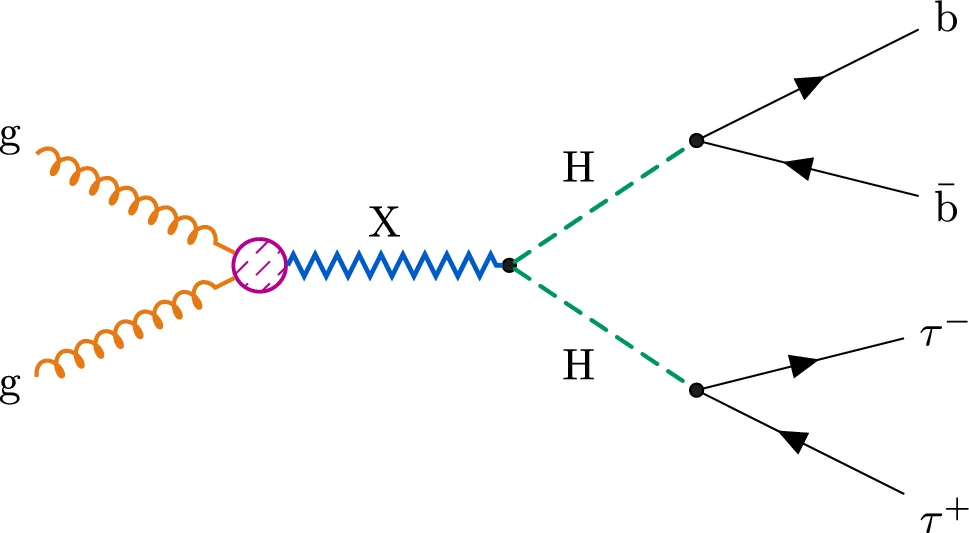
A representative diagram for the production of a spin-0 radion or a spin-2 graviton X, which decays into two SM Higgs bosons. One Higgs boson decays into a $\hbox {b}\bar{\text {b}}$ pair and the other into a $\tau ^{+}\tau ^{-}$ pair

The observation of the H boson has opened new avenues for probing BSM scenarios. In fact, many BSM theories predict the existence of heavy resonances (X) with masses exceeding 1$\,\text {Te}\hspace{-.08em}\text {V}$   that can decay into a pair of Higgs bosons (HH). The production of Higgs boson pairs is a crucial, but rare, process in the SM. If resonant HH production occurs through BSM physics, it can significantly enhance the cross section. An example of a well-motivated theoretical framework predicting such enhancements is the warped extra dimensions model proposed by Randall and Sundrum (RS) [6, 7], which leads to the existence of new neutral, spin-0 radions  [8–10] and spin-2 Kaluza–Klein gravitons [11, 12]. The analysis presented in this paper is designed to be model independent, with a single strategy for both spin-0 and spin-2 resonances, each decaying into a pair of Higgs bosons in the $\text {b}\bar{\hbox {b}}\,\tau ^{+}\tau ^{-}$ final state. A representative diagram illustrating the production of such a resonance is shown in Fig. 1.

This search targets resonance masses between 1 and 4.5$\,\text {Te}\hspace{-.08em}\text {V}$   using the CMS proton–proton (pp) collision data sample collected at a center-of-mass energy of $13\,\text {Te}\hspace{-.08em}\text {V}$ during the years 2016–2018. A previous CMS search for heavy resonant HH production in the same final state [13] is based on a smaller data set, using only the 2016 pp  collision data, corresponding to 35.9$\,\text {fb}^{-1}$. The observed data in that analysis are consistent with SM expectations, and upper limits are set at 95% confidence level ($\text {CL}$) on the production cross section for spin-0 and spin-2 resonance masses between 0.9 and 4.0$\,\text {Te}\hspace{-.08em}\text {V}$. The analysis performed by the ATLAS Collaboration in the boosted topology is based on the pp data collected in 2015–2018 and covers resonance masses from 1 to 3$\,\text {Te}\hspace{-.08em}\text {V}$  [14]. No significant excess is observed in that search either, and 95% $\text {CL}$   exclusion limits are set in the signal range of 1–3$\,\text {Te}\hspace{-.08em}\text {V}$. A separate ATLAS search on the same data set targets spin-0 resonances, decaying to HH, with masses from 251$\,\text {Ge}\hspace{-.08em}\text {V}$ to 1.6$\,\text {Te}\hspace{-.08em}\text {V}$  [15]. While this ATLAS search primarily targets the resolved topology, it covers the same signal mass range as the present analysis, that is, between 1 and 1.6$\,\text {Te}\hspace{-.08em}\text {V}$. The data are also found to be compatible with the background-only hypothesis, with the largest deviation at a mass of 1$\,\text {Te}\hspace{-.08em}\text {V}$, corresponding to local and global significances of 3.1 and 2.0 standard deviations, respectively.

This paper focuses on the fully hadronic ($\tau _h\tau _h$) and the mixture of one leptonic and one hadronic decay ($\ell \tau _h$) of the $\tau ^{+}\tau ^{-}$ pair in resonant Higgs boson pair production, where one H boson decays into $\hbox {b}\bar{\text {b}}$ and the other into $\tau ^{+}\tau ^{-}$. The individual hadronically-decaying tau leptons are denoted as $\uptau _\text {h}$, while ${{\ell }}$ denotes an electron or muon. In addition to ${\text {X}} \rightarrow {\text {HH}} \rightarrow \hbox {b}\bar{\text {b}}\, \tau ^{+}\tau ^{-}$, other resonant HH processes that can contribute to the same final states are also included as signal, namely ${\text {X}} \rightarrow {\text {HH}} \rightarrow \hbox {b}\bar{\text {b}}\,{\text {W}} {\text {W}} \rightarrow \hbox {b}\bar{\text {b}}\,\ell ^{\ddagger }{\upnu } \text {q} \text {q}$ and ${\text {X}} \rightarrow {\text {HH}} \rightarrow \hbox {b}\bar{\text {b}}\, {\text {V}} {\text {V}} \rightarrow \hbox {b}\bar{\text {b}}\, \ell ^{\ddagger }\ell ^{\ddagger }{\upnu } {\upnu }$. Here, $\ell ^{\ddagger }$ denotes a charged lepton of any flavor (*e*, μ, or τ), $\text {q}$ represents a quark, and V denotes a massive SM vector boson (W or Z), as used throughout this paper. In the simulated signal samples of ${\text {H}} \rightarrow \tau ^{+}\tau ^{-}$, the ${\tau }$  leptons decay inclusively according to their SM branching fractions, while for ${\text {H}} \rightarrow {\text {W}} {\text {W}}$ and ${\text {H}} \rightarrow {\text {Z}} {\text {Z}}$ decays, we restrict the V decays to the final states listed above, i.e., ${\text {W}} {\text {W}} \rightarrow \ell ^{\ddagger }{\upnu } \ell ^{\ddagger }{\upnu }$, $\ell ^{\ddagger }{\upnu } \text {q} \text {q}$, and ${\text {Z}} {\text {Z}} \rightarrow \ell ^{\ddagger }\ell ^{\ddagger }{\upnu } {\upnu }$. Therefore, the corresponding SM branching fractions [16, 17] of W and Z bosons to these final states are taken into account when calculating the signal yields.

This analysis assumes a narrow resonance width, i.e., the natural width of the resonance (1$\,\text {Me}\hspace{-.08em}\text {V}$) is significantly smaller than the experimental resolution (around 10% of the mass of the resonance). The two bottom quarks from the Higgs boson decay are produced with a small angular separation and often merge into a single jet after hadronization. These jets are identified with a graph convolutional neural network called the ParticleNet jet tagger [18]. In this Lorentz-boosted regime, the decay products of the $\boldsymbol{\uptau }$ lepton pair, whether $\ell \tau _h$ or $\tau _h\tau _h$, tend to overlap spatially, making di-$\boldsymbol{\uptau }$ reconstruction particularly challenging. The reconstruction of such boosted $\boldsymbol{\uptau }$ leptons was first addressed in a similar CMS search using 8$\,\text {Te}\hspace{-.08em}\text {V}$   pp   collision data [19]. In this analysis, we enhance the sensitivity of these methods by employing a dedicated DeepTau [20] based tagger, optimized specifically for the reconstruction of boosted $\boldsymbol{\uptau }$ leptons. The SM processes with the largest contributions to the background are $\text {t}\bar{\text {t}}$+jets (top quark-antiquark pair production), $\text {W}$+jets, and $\text {Z}$+jets production, although the precise composition depends on the specific channel under consideration.

The structure of this paper is as follows: Sect. 2 provides an overview of the CMS detector, while Sect. 3 describes the CMS data set and the simulation of physics processes. Section 4 details the object and event reconstruction, followed by Sect. 5, which outlines the event selection and categorization. The methods used for background modeling are discussed in Sect. 6, and systematic uncertainties are addressed in Sect. 7. Finally, the results are presented in Sect. 8, and the paper is summarized in Sect. 9. Tabulated results for this analysis are provided in HEPData [21].

## The CMS detector

The central feature of the CMS apparatus is a superconducting solenoid of 6m internal diameter, providing a magnetic field of 3.8T. Within the solenoid volume are a silicon pixel and strip tracker, a lead tungstate crystal electromagnetic calorimeter (ECAL), and a brass and scintillator hadron calorimeter (HCAL), each composed of a barrel and two endcap sections. Forward calorimeters extend the pseudorapidity (η) coverage provided by the barrel and endcap detectors. Muons are detected in gas ionization chambers interleaved with the layers of the steel flux-return yoke outside the solenoid. A more detailed description of the CMS detector, together with a definition of the coordinate system used and the relevant kinematic variables, can be found in Refs. [22] and [23].

Events of interest are selected using a two-tiered trigger system [24–26]. The first level, composed of custom hardware processors, uses information from the calorimeters and muon detectors to select events at a rate of around 100kHz within a time interval of less than 4$\,\mu \text {s}$. The second level, known as the high-level trigger, consists of a farm of processors running a version of the full event reconstruction software optimized for fast processing, and reduces the event rate to a few kHz before data storage.

## Data and simulated samples

This search utilizes data recorded by the CMS detector from 2016 to 2018, corresponding to an integrated luminosity of 138$\,\text {fb}^{-1}$. Events are selected using a set of triggers that require either missing transverse momentum ($p_{\textrm{T}} ^\text {miss}$) or missing hadronic transverse momentum ($H_{\textrm{T}}^{\text {miss}}$) to exceed 110$\,\text {Ge}\hspace{-.08em}\text {V}$, often in conjunction with additional criteria, such as the presence of a jet with $p_{\textrm{T}} > 80\,\text {Ge}\hspace{-.08em}\text {V}$. The primary vertex (PV) [27] is defined as the vertex with the largest $p_{\textrm{T}} ^{2}$ sum of the physics objects originating from that vertex. Here, the physics objects are the charged leptons, the AK4 jets, and the $p_{\textrm{T}} ^\text {miss}$. The missing transverse momentum vector $\vec {p}_{\textrm{T}}^{\text {miss}}$ is defined as the negative vector sum of the momentum of all reconstructed particle candidates associated with the PV, projected onto the plane perpendicular to the beam direction. The missing transverse momentum $p_{\textrm{T}} ^\text {miss}$ is defined as the magnitude of $\vec {p}_{\textrm{T}}^{\text {miss}}$. The observable $H_{\textrm{T}}^{\text {miss}}$ is defined as the magnitude of the vectorial $p_{\textrm{T}}$ sum of all jets with $p_{\textrm{T}} >30\,\text {Ge}\hspace{-.08em}\text {V}$ and |η|<3.0. The particle and jet reconstruction are described in detail in Sect. 4.

Monte Carlo (MC) simulated samples of the main SM backgrounds and of the signal processes are used to optimize the event selection and to improve the background estimation. Simulated events of $\text {t}\bar{\text {t}}+$jets production and single top quark processes (*t*-, *s*-, and ${\text {t}} {\text {W}}$-channels) are generated at next-to-leading order (NLO) in quantum chromodynamics (QCD) using the powheg v1 and v2 [28, 29] event generators. For the $\text {t}\bar{\text {t}}+$jets process, the top quark $p_{\textrm{T}}$ distribution is reweighted to match next-to-next-to-leading order (NNLO) theoretical computations [30]. Samples of $\text {Z}$+jets and $\text {W}$+jets are generated at leading order (LO) using MadGraph5_amc@nlo v2.6.5 [31] with the MLM prescription [32] for matching jets from the matrix element calculation to the parton shower. Dedicated electroweak [33–38] and QCD (calculated with MadGraph5_amc@nlo) NLO/LO *k*-factors, parameterized as functions of the generated boson $p_{\textrm{T}}$, are applied to $\text {Z}$+jets and $\text {W}$+jets events. The QCD multijet events are simulated at LO either using the pythia  [39] or MadGraph5_amc@nlo event generators, with MLM matching [40] for the latter. Other processes with smaller contributions, such as diboson production, are generated at NLO using either MadGraph5_amc@nlo with FxFx [41] matching or powheg v2. All background samples are normalized using the most precise cross section evaluations available, which generally are calculated at NLO or NNLO.

Signal samples of narrow-width $\varGamma _{{\text {X}}} = 1\,\text {Me}\hspace{-.08em}\text {V}$ spin-0 and spin-2 resonances, produced via gluon fusion and decaying to HH, are simulated at LO using MadGraph5_amc@nlo. The spin-0 (spin-2) signal is modeled as a radion-like scalar (graviton-like) resonance, with spin correlations handled through the corresponding matrix elements. Resonance masses range from 1 to 4.5$\,\text {Te}\hspace{-.08em}\text {V}$. For spin-0 resonances, mass points are spaced by 200$\,\text {Ge}\hspace{-.08em}\text {V}$ between 1 and 2$\,\text {Te}\hspace{-.08em}\text {V}$, and by 500$\,\text {Ge}\hspace{-.08em}\text {V}$ above 2$\,\text {Te}\hspace{-.08em}\text {V}$. For spin-2 resonances, the spacing is 250$\,\text {Ge}\hspace{-.08em}\text {V}$ between 1 and 2$\,\text {Te}\hspace{-.08em}\text {V}$, and 500$\,\text {Ge}\hspace{-.08em}\text {V}$ above 2$\,\text {Te}\hspace{-.08em}\text {V}$.

The initial-state partons are modeled with the NNPDF 3.1 NNLO [42] parton distribution function (PDF) set. Parton showering and hadronization are handled by pythia v8.230 [39] using the CP5 tune [43] for most samples. All signal and background samples are processed using Geant4  [44] to provide a full simulation of the CMS detector, including simulation of the triggers. The effects of additional pp interactions in the same or adjacent bunch crossings, referred to as pileup, are included in all simulated samples. To match the simulated distribution of pileup interactions with the one observed in data, a reweighting procedure is implemented. Correction factors are derived and applied to the simulated samples to match the trigger efficiencies measured in data. Additional corrections are applied to cover residual differences between data and simulation that arise from the lepton identification and reconstruction efficiencies, as well as from identification efficiencies for jets identified to originate from b quarks.

## Event reconstruction

A particle-flow (PF) algorithm [45] reconstructs and identifies each individual particle through an optimized combination of information from the various elements of the CMS detector. The energy of each electron is determined from a combination of the electron momentum as measured by the tracker, the energy of the corresponding ECAL cluster, and the energy sum of all bremsstrahlung photons spatially compatible with originating from the electron track. The energies of muons are obtained from the curvature of the corresponding track. The energies of charged hadrons are determined from a combination of their momenta measured in the tracker and the matching ECAL and HCAL energy deposits, corrected for zero-suppression effects and for the response function of the calorimeters to hadronic showers. Finally, the energies of neutral hadrons are obtained from the corresponding corrected ECAL and HCAL energies.

The identified particles are clustered into jets using the anti-$k_{\textrm{T}}$ algorithm [46], implemented in the FastJet package [47]. Two distance parameters are used in the analysis, 0.4 and 0.8, yielding jet collections referred to as AK4 and AK8 jets, respectively. The PF candidates and AK4 jets are used in the definitions of $p_{\textrm{T}} ^\text {miss}$ and $H_{\textrm{T}}^{\text {miss}}$. The AK4 jets are used primarily to reject or select events with top quarks, while the larger AK8 jets are used to identify and contain hadronically decaying H boson candidates.

Additional selection criteria are applied to each event to remove spurious jet-like features originating from isolated noise patterns in certain HCAL regions [48]. The AK4 and AK8 jets must have $p_{\textrm{T}} >20\,\text {Ge}\hspace{-.08em}\text {V}$ and $p_{\textrm{T}} >200\,\text {Ge}\hspace{-.08em}\text {V}$, respectively and |η|<2.4, to be considered in the subsequent steps of the analysis.

To improve the stability of the jet mass and substructure variables in events with high pileup, the pileup-per-particle identification (PUPPI) algorithm [49] is applied to the AK8 jets used to identify hadronic boson decays. The PUPPI algorithm uses the local distribution of particles, event pileup properties, and tracking information to compute a weight describing the likelihood for each particle to originate from a pileup interaction. The weight is used to rescale the particle four-momentum, superseding the need for further jet-based pileup corrections.

Large AK8 jets originating from the dominant $\hbox {b}\bar{\text {b}}$ decays of H bosons are likely to have two displaced vertices because of the long lifetime and large mass of the b quarks. A graph convolutional neural network, ParticleNet [18], is used to distinguish H boson decays into $\hbox {b}\bar{\text {b}}$ from background jets by leveraging the properties of jet PF constituents as input features. As with all heavy-flavor jet classifiers, displaced tracks and secondary vertices are among the most important features. The multiclass ParticleNet algorithm provides several output scores, each ranging from 0 to 1, representing the probability that a jet originates from a specific process, such as H boson decay to b quarks ($P({\text {H}} \rightarrow \hbox {b}\bar{\text {b}})$), or a light-flavor quark or gluon ($P(\text {QCD})$). Following the standard approach for constructing a binary discriminant to distinguish H boson jets from the dominant QCD multijet background [50], the ParticleNet score is defined as $P({\text {H}} \rightarrow \hbox {b}\bar{\text {b}}) / ( P({\text {H}} \rightarrow \hbox {b}\bar{\text {b}}) + P(\text {QCD}))$. In addition to classification, ParticleNet performs a regression task to estimate the mass of the AK8 jet, referred to in this work as the ParticleNet regressed mass $M_{\text {H}(\text {b}{\bar{\text {b}}})}$. In this analysis, we define a signal-enriched region (SR), defined as $100< M_{\text {H}(\text {b}{\bar{\text {b}}})} < 150\,\text {Ge}\hspace{-.08em}\text {V}$, and a second region, called the sideband (SB), defined as $M_{\text {H}(\text {b}{\bar{\text {b}}})} < 100\,\text {Ge}\hspace{-.08em}\text {V}$ or $M_{\text {H}(\text {b}{\bar{\text {b}}})} > 150\,\text {Ge}\hspace{-.08em}\text {V}$, enriched in background processes.

The AK4 jets originating from b quarks are identified using the DeepJet algorithm [51], a deep neural network that combines secondary vertex information, track-based variables, and PF jet constituents (neutral and charged-particle candidates). Events are vetoed if they contain at least one b-tagged AK4 jet that does not overlap with the selected AK8 jet or the leptons in the event, to further suppress the $\bar{\text {t}}$ background.

A series of dedicated algorithms are used to reconstruct and identify H bosons decaying to $\tau ^{+}\tau ^{-}$ pairs with either $\tau _h\tau _h$ or $\ell \tau _h$ signatures. These procedures are described in the rest of this section.

Highly collimated $\tau _h\tau _h$ or $\ell \tau _h$ pairs are quite challenging to reconstruct and require the use of a special boosted reconstruction algorithm [52]. The procedure begins by using the Cambridge–Aachen algorithm [53] with a distance parameter of 0.8 to identify jets with a large cone size, called CA8 jets. For each CA8 jet with $p_{\textrm{T}} >100\,\text {Ge}\hspace{-.08em}\text {V}$, the last step of the clustering is retracted, resulting in two subjets. If these subjets are found to have $p_{\textrm{T}} >10\,\text {Ge}\hspace{-.08em}\text {V}$ and satisfy the mass drop condition, which requires $\max (m_\mathrm {subjet\,1}, m_\mathrm {subjet\,2})/m_\mathrm {CA8~jet} <2/3$, the two subjets are used as inputs to the standard ${\tau }$ lepton reconstruction, the “hadron-plus-strips” (HPS) algorithm [54], to build the $\uptau _\text {h}$ candidates. Here, $m_\mathrm {subjet\,1}$ and $m_\mathrm {subjet\,2}$ are the masses of the two subjets, and $m_\mathrm {CA8~jet}$ is the mass of the original CA8 jet. If the $p_{\textrm{T}}$ and mass drop conditions are not met, the unclustering and identification procedures are repeated iteratively for the most energetic subjet. We will refer to $\boldsymbol{\uptau }$ leptons reconstructed in this fashion as boosted $\uptau _\text {h}$.

The boosted reconstruction algorithm is not well suited for H bosons with lower Lorentz boost, as is typical of lower resonance mass signal hypotheses around 1 to 1.5$\,\text {Te}\hspace{-.08em}\text {V}$. To cover this topology, we instead reconstruct single $\uptau _\text {h}$ candidates using AK4 jets as inputs to the same HPS algorithm. These are referred to as standard $\uptau _\text {h}$.

The final selection of $\uptau _\text {h}$ candidates, whether boosted or standard, requires |η|<2.5 and $p_{\textrm{T}} > 20\,\text {Ge}\hspace{-.08em}\text {V}$, along with a convolutional neural network based tagger score above a specified threshold corresponding to per-$\uptau _\text {h}$ signal efficiency greater than 95%. The identification of boosted $\uptau _\text {h}$ candidates was developed specifically for this analysis and is based on the DeepTau architecture originally designed for standard $\boldsymbol{\uptau }$ leptons. This tagger, referred to as BoostedDeepTau throughout the article, is trained to distinguish genuine boosted $\boldsymbol{\uptau }$ leptons from background jets by leveraging both high-level features derived from the boosted $\boldsymbol{\uptau }$ reconstruction and low-level detector information. Among its important inputs are a global event-level variable related to the average energy deposition in the event and 42 high-level variables that capture the kinematic properties, isolation, and finite lifetime signature of boosted $\boldsymbol{\uptau }$ leptons. Many of these variables have also proven effective in previously developed $\boldsymbol{\uptau }$ identification algorithms, such as a discriminator based on a boosted decision tree (MVA Iso) [52].

The training of the BoostedDeepTau algorithm is performed using simulated samples. Genuine boosted $\boldsymbol{\uptau }$ leptons are taken from simulated spin-0 signal samples, while the dominant backgrounds—QCD multijet, $\text {t}\bar{\text {t}}$, and Drell–Yan processes—are included to ensure comprehensive coverage of the relevant phase space. The training samples include various mass points and are shuffled to balance the boosted $\boldsymbol{\uptau }$ lepton population across $p_{\textrm{T}}$, η, and other relevant variables. The resultant BoostedDeepTau tagger shows a discrimination power that is a factor 2–4 better than MVA Iso evaluated on boosted $\boldsymbol{\uptau }$ leptons for $p_{\textrm{T}} <100\,\text {Ge}\hspace{-.08em}\text {V}$ and more than a factor of 10 better for $p_{\textrm{T}} >100\,\text {Ge}\hspace{-.08em}\text {V}$. Additional information about the BoostedDeepTau algorithm can be found in Ref. [55].

Electrons from leptonic tau decays are reconstructed in the region |η|<2.5 by matching energy deposits in the ECAL with tracks reconstructed in the tracker [56]. Electron identification is based on the distribution of energy deposited along the electron trajectory, and the direction and momentum of the track in the inner tracker. Additional requirements are applied to remove electrons produced through photon conversions. Electrons are also required to be isolated from other particles in the detector, by imposing an upper threshold on the relative isolation parameter. The electron isolation parameter is defined as the magnitude of the $p_{\textrm{T}}$ sum of all the PF candidates (excluding the electron) within ΔR<0.3 around the electron direction, after the contributions from pileup and particles associated with reconstructed $\uptau _\text {h}$ candidates within the isolation cone have been removed. The resulting sum is then divided by the electron $p_{\textrm{T}}$, yielding a corrected relative isolation variable $I_\text {rel}^{\text {e}}$, which is required to satisfy $I_\text {rel}^{\text {e}} < 0.112 + 0.506/p_{\textrm{T}}$ in the ECAL barrel (|η|<1.479) and $I_\text {rel}^{\text {e}} < 0.108 + 0.963/p_{\textrm{T}}$ in the ECAL endcaps (1.479<|η|<2.5), with $p_{\textrm{T}}$ in $\,\text {Ge}\hspace{-.08em}\text {V}$.

Muons from leptonic tau decays are reconstructed within the acceptance of the CMS muon system, |η|<2.4, using information from both the muon spectrometer and the silicon tracker [57]. Muon candidates are identified based on the compatibility of tracks reconstructed in the silicon tracker with tracks reconstructed from a combination of hits in both the tracker and the muon detector. In addition, the trajectory is required to be compatible with originating from the primary vertex, and to have a sufficient number of hits in the tracker and muon systems. Muons are required to be isolated by applying a relative isolation criterion. Specifically, the scalar sum of the $p_{\textrm{T}}$ of all PF candidates within a cone of ΔR<0.4 around the muon direction—excluding the muon itself—is computed. Contributions from particles associated with reconstructed $\uptau _\text {h}$ candidates within the isolation cone are subtracted. The resulting sum is then divided by the muon $p_{\textrm{T}}$, yielding a corrected relative isolation variable $I_\text {rel}^{\upmu }$, which is required to satisfy $I_\text {rel}^{\upmu } < 0.25$. This procedure is very similar to that followed for the electrons.

The standard (boosted) $\uptau _\text {h}$ candidates are either combined with another standard (boosted) $\uptau _\text {h}$ candidate or with a leptonic $\boldsymbol{\uptau }$ decay identified as an electron or a muon, to form $\tau ^{+}\tau ^{-}$ pairs. Both standard $\uptau _\text {h}$ and boosted $\uptau _\text {h}$ candidates are required to be distinct from the selected AK8 jet in the event.

## Event selection

As mentioned earlier in Sect. 3, events are selected using a suite of $p_{\textrm{T}} ^\text {miss}$ and $H_{\textrm{T}}^{\text {miss}}$ triggers. For the heavy parent resonance masses considered in this analysis, the H bosons are produced with large Lorentz boosts. As a result, the neutrinos from the $\tau ^{+}\tau ^{-}$ decay carry substantial momentum, and signal events are therefore expected to exhibit large $p_{\textrm{T}} ^\text {miss}$, ensuring high efficiency of the $p_{\textrm{T}} ^\text {miss}$ and $H_{\textrm{T}}^{\text {miss}}$ triggers. A stringent offline requirement of $p_{\textrm{T}} ^\text {miss} >180\,\text {Ge}\hspace{-.08em}\text {V}$ is applied after the full event reconstruction, to ensure a stable trigger efficiency and to suppress the background contribution from multijet events. The efficiency of the trigger for events satisfying the offline event selection, measured in an independent sample of events selected with muon triggers, is found to be greater than 95%, with an uncertainty of 1.1%.

All events are required to contain one H boson candidate decaying to either ${{\ell }} \uptau _\text {h}$ or $\uptau _\text {h} \uptau _\text {h}$. If multiple candidate pairs are possible, the pair with the highest visible $p_{\textrm{T}}$ is selected. The other H boson candidate decaying to $\hbox {b}\bar{\text {b}}$ is reconstructed as an AK8 jet that does not overlap with the $\tau ^{+}\tau ^{-}$ system. The same AK8 jet kinematic selection and identification requirements are applied in both channels. If multiple AK8 jet candidates are present, the jet with the highest $p_{\textrm{T}}$ is selected. The ${\text {H}} \rightarrow \text {b}\bar{\hbox {b}}$ candidate jet is also required to satisfy the ParticleNet $\hbox {b}\bar{\text {b}}$ tagging requirement corresponding to a working point with 80% efficiency for selecting signal jets from ${\text {H}} \rightarrow \text {b}\bar{\hbox {b}}$ decays. This criterion significantly suppresses background from QCD multijet events and jets produced in association with vector bosons. Events with top quark pairs or single top quarks are suppressed by vetoing events with b-tagged AK4 jets that do not overlap with the AK8 ${\text {H}} \rightarrow \hbox {b}\bar{\text {b}}$ candidate and are distinct from the identified ${\text {H}} \rightarrow \tau ^{+}\tau ^{-}$ objects.

Additional selection criteria are applied to suppress SM contributions, including those from meson and baryon resonances, $\text {W}$+jets, $\text {t}\bar{\text {t}}$, and single top quark production. To reduce contamination from misidentified $\boldsymbol{\uptau }$ leptons in $\text {W}$+jets events, the angular separation $\varDelta R _{\tau ^{+}\tau ^{-}}$ between the two $\boldsymbol{\uptau }$ candidates is required to be less than 1.5. Additionally, we require the difference in the azimuthal angle between the AK8 jet, identified as the H boson through the ${\text {H}} \rightarrow \hbox {b}\bar{\text {b}}$ decay, and $\vec {p}_{\textrm{T}}^{\text {miss}}$ to be greater than 1. This requirement ensures that the Higgs boson candidate and the missing transverse momentum are well separated in the transverse plane, typically appearing on opposite sides of the event, as expected in signal-like topologies. To reduce contributions from low-$p_{\textrm{T}}$ radiation, the soft-drop (SD) [58] jet mass of the ${\text {H}} \rightarrow \hbox {b}\bar{\text {b}}$ candidate jet is required to be greater than 30$\,\text {Ge}\hspace{-.08em}\text {V}$. Furthermore, the visible mass of the H boson decaying into $\tau ^{+}\tau ^{-}$ must be greater than 20$\,\text {Ge}\hspace{-.08em}\text {V}$ to suppress low-mass and quarkonium backgrounds.

An algorithm called FastMTT [59], based on a dynamical likelihood approach, is used to reconstruct the four momentum of the di-$\boldsymbol{\uptau }$ system, including the missing information from neutrinos. This method incorporates the measured momentum of the visible $\boldsymbol{\uptau }$ decay products, the reconstructed $p_{\textrm{T}} ^\text {miss}$, and its resolution to enhance the reconstruction accuracy. To speed up the computation, the algorithm applies the collinear approximation [60], which assumes that the neutrinos from $\boldsymbol{\uptau }$ decays are collinear with the visible decay products. The ${\text {H}} \rightarrow \hbox {b}\bar{\text {b}}$ candidate four-momentum is formed using kinematic variables obtained from the standard AK8 jet reconstruction algorithm, to which energy and resolution corrections are applied to match the response in data.

**Fig. 2:**
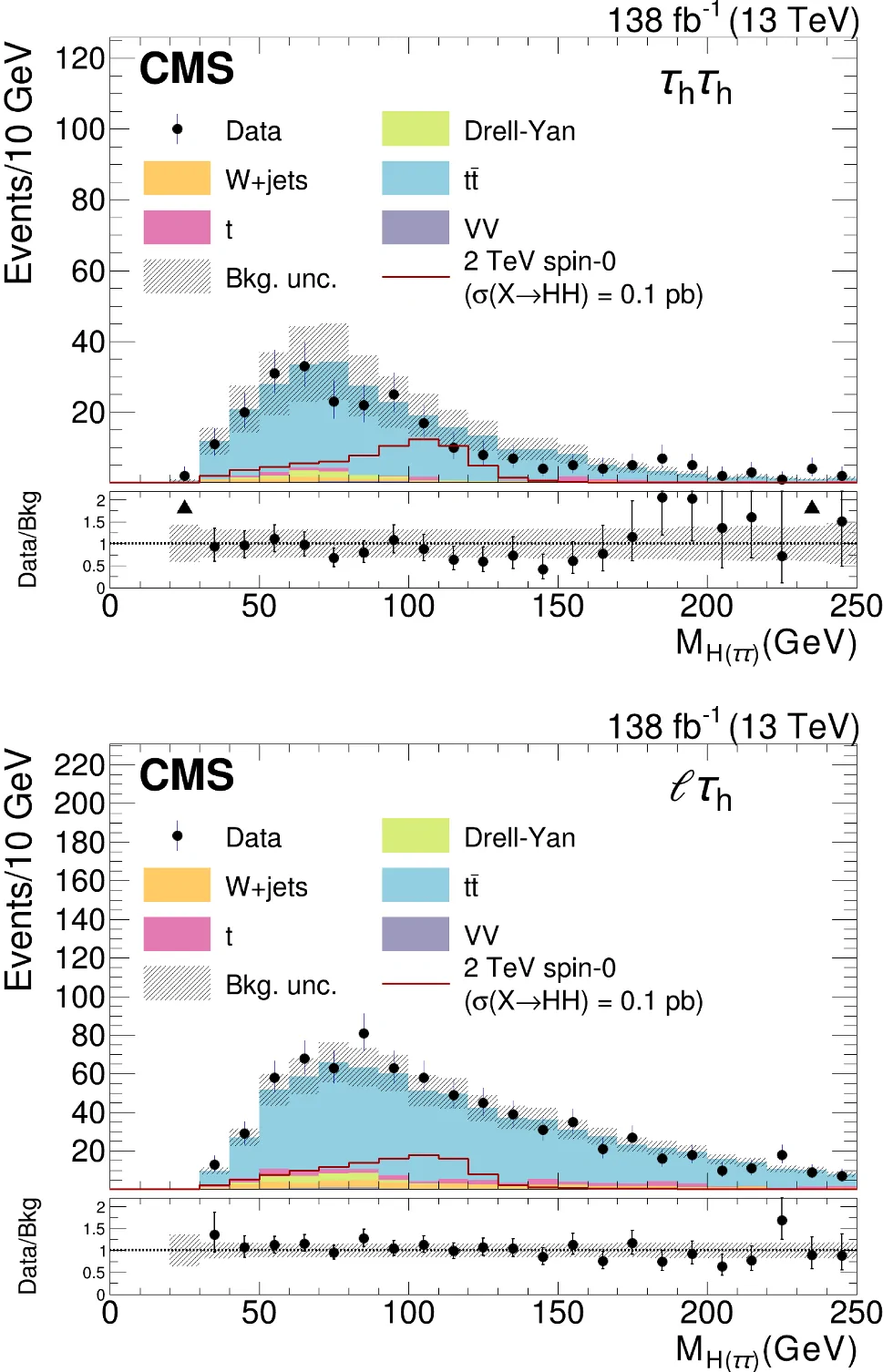
Distribution of the invariant mass of the di-$\boldsymbol{\uptau }$ system, reconstructed with the FastMTT algorithm, after the full event selection in the $\tau _h\tau _h$ (upper) and $\ell \tau _h$ (lower) channels. The data (solid circles) are compared to the background simulation (filled histograms), where the gray bands represent the total background uncertainty, obtained from the post-fit values of the dominant systematic uncertainties and the statistical uncertainties in the simulated samples. The $\textrm{X}\rightarrow \textrm{HH}$ signal simulation (solid red line) is overlaid and normalized to $\sigma (\textrm{X}\rightarrow \textrm{HH})=0.1~{\text {pb}}$ for illustration. The ratio between the data and the total expected background contribution is shown in the lower panel, where a solid black triangle indicates those bins where the ratio exceeds the axis range

**Fig. 3:**
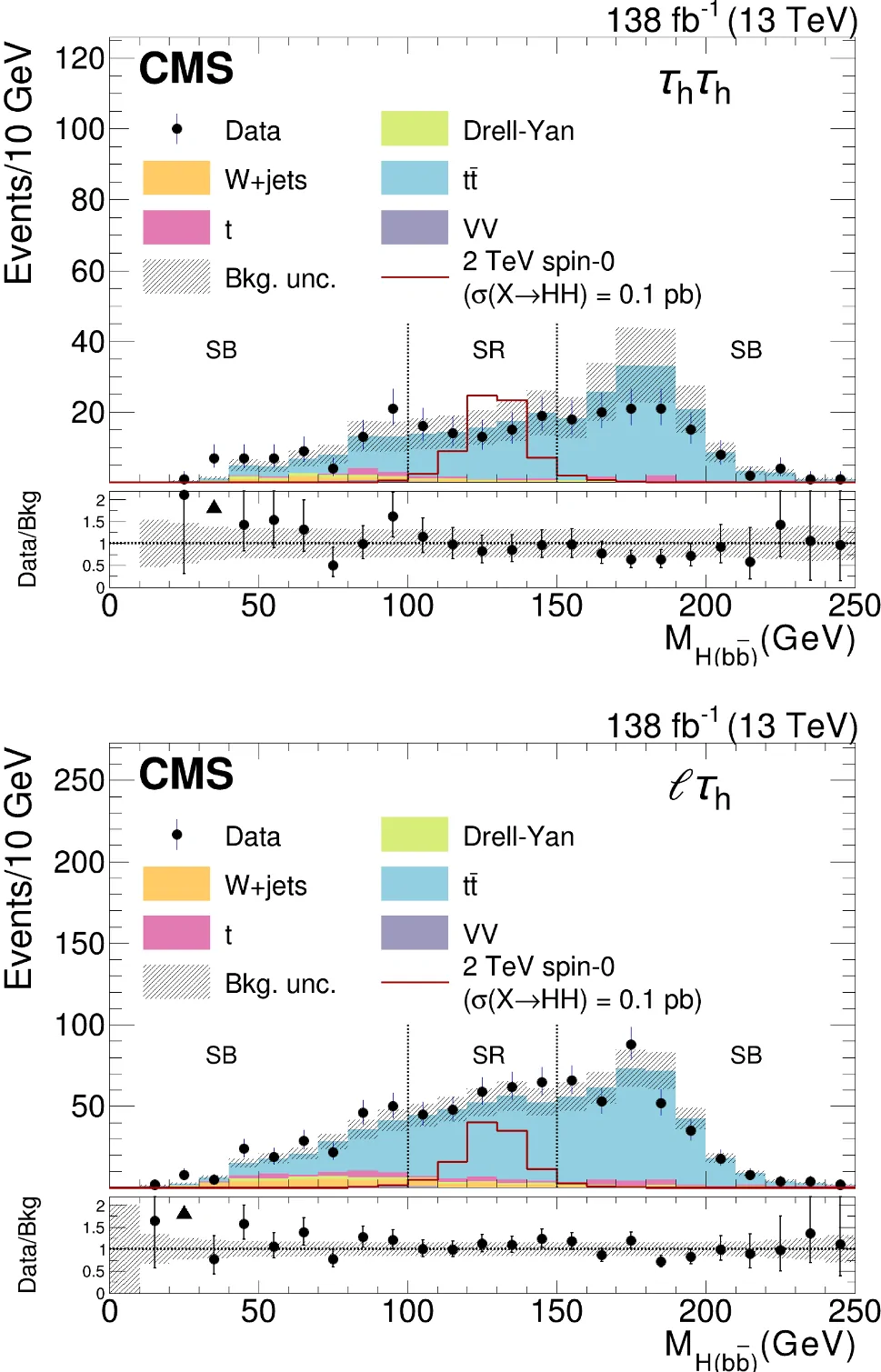
Distribution of $M_{\text {H}(\text {b}{\bar{\text {b}}})}$ obtained from the leading AK8 jet in the event after the full event selection for the $\tau _h\tau _h$ (upper) and $\ell \tau _h$ (lower) channels. The signal-enriched region (SR) is defined as $100< M_{\text {H}(\text {b}{\bar{\text {b}}})} < 150\,\text {Ge}\hspace{-.08em}\text {V}$. The sideband (SB) is immediately adjacent to the SR, on either side. The data (solid circles) are compared to the background simulation (filled histograms), where the gray bands represent the total background uncertainty, obtained from the post-fit values of the dominant systematic uncertainties and the statistical uncertainties in the simulated samples. The $\textrm{X}\rightarrow \textrm{HH}$ signal simulation (solid red line) is overlaid and normalized to $\sigma (\textrm{X}\rightarrow \textrm{HH})=0.1~{\text {pb}}$ for illustration. The ratio between the data and the total expected background contribution is shown in the lower panel, where a solid black triangle indicates those bins where the ratio exceeds the axis range

The reconstructed resonance candidate mass $M_{{\text {X}}}$, defined as the invariant mass of the tagged ${\text {H}} \rightarrow \hbox {b}\bar{\text {b}}$ candidate jet and the FastMTT reconstructed di-$\boldsymbol{\uptau }$ system, is required to be larger than 750$\,\text {Ge}\hspace{-.08em}\text {V}$ to ensure full trigger and reconstruction efficiencies. All selections, except for the ParticleNet $\hbox {b}\bar{\text {b}}$-tagging and the AK4 b-tagged event veto, are collectively referred to as the “pre-selection” in this analysis. The distributions of important kinematic variables for the full event selection are shown in Figs. 2 and 3.

The full event selection efficiency, including the trigger selections, for spin-0 (spin-2) resonances ranges from 2% (2.7%) to 13.5% (14.4%) in the $\uptau _\text {h} \uptau _\text {h}$ channel and from 4% (5.4%) to 11% (11.8%) in the ${{\ell }} \uptau _\text {h}$ channel, depending on the mass of the resonance. Additionally, events from the ${\text {HH}} \rightarrow {\hbox {b}}\bar{\text {b}}\,\text {VV}$ processes contribute approximately 20% of the total yield of the ${\text {HH}} \rightarrow \hbox {b}\bar{\text {b}}\,\tau ^{+}\tau ^{-}$ signal. Around 90% of the selected ${\text {HH}} \rightarrow \hbox {b}\bar{\text {b}}\,\text {VV}$ events originate from genuine ${{\ell }} \uptau _\text {h}$ or $\uptau _\text {h} \uptau _\text {h}$ final states.

To extract a potential resonant HH signal, a maximum likelihood fit is performed using the $M_{{\text {X}}}$ distributions in data in the $\ell \tau _h$ and $\tau _h\tau _h$ channels. These fits are performed using the CMS statistical analysis tool Combine [61], which is based on the RooFit [62] and RooStats [63] frameworks. Accurate modeling of the major backgrounds is essential for the signal extraction. The strategy for estimating this is described in the following section.

## Background estimation

After the event pre-selection, the leading SM background contributions in the different regions are from $\text {t}\bar{\text {t}}$, $\text {Z}$+jets, and $\text {W}$+jets production. Following the full event selection, the $\text {t}\bar{\text {t}}$ process becomes the dominant background, as shown in Figs. 2 and 3. The QCD multijet contribution is negligible owing to the lepton requirement in the $\ell \tau _h$ channel, and the combined effect of the BoostedDeepTau/DeepTau and the ParticleNet-based AK8 ${\text {H}} \rightarrow \hbox {b}\bar{\text {b}}$ selections in the $\tau _h\tau _h$ channel.

The SR and SB regions are defined using the reconstructed $M_{\text {H}(\text {b}{\bar{\text {b}}})}$ distribution, as shown in Fig. 3. The $M_{\text {H}(\text {b}{\bar{\text {b}}})}$ distribution is used only to categorize events into regions, whereas the final discriminant used in the fit is the $M_{{\text {X}}}$ distribution. We use the data in the SB to constrain the normalization and the $M_{{\text {X}}}$ shape of the top quark background in the SR, where the top quark background in both the SR and the SB combines the contributions from $\text {t}\bar{\text {t}}$ and single top quark production. This is achieved by introducing unconstrained, bin-by-bin multiplicative parameters for each bin of the $M_{{\text {X}}}$ distribution. These parameters simultaneously adjust the yield of the top quark background per $M_{{\text {X}}}$ bin in both the SR and SB for the corresponding channel during the fit.

## Systematic uncertainties

**Table 1:** Summary of the dominant systematic uncertainty sources and the typical size of their inferred variation in the resonance yield. Unless otherwise specified, the impact is the same for the $\tau _h\tau _h$ and $\ell \tau _h$ channels. Sources with an overall impact of less than 1% on the resonance yield, such as electron or muon reconstruction and identification, are not listed in the table

Systematic source	Variation
Boosted $\uptau _\text {h}$ lepton identification	20% for $\tau _h\tau _h$, 10% for $\ell \tau _h$
$\uptau _\text {h}$ energy scale	10%
Renormalization and factorization scales	5–10%
PDF uncertainties	5–7%
ParticleNet AK8 $\hbox {b}\bar{\text {b}}$ tagging	5% (signal jets)
Jet energy scale and resolution	2–5%
Pileup modelling	1–5%
Electroweak and QCD *k*-factors for $\textrm{V}+$jets	1–5%
Integrated luminosity	1.6%
$p_{\textrm{T}} ^\text {miss}$ trigger	1.1%

Various sources of systematic uncertainties are taken into account, in most cases affecting both the signal and the background. We distinguish between two types of systematic uncertainties: those that affect only the overall normalization of a process and those that also impact the shape of the reconstructed resonance mass spectrum, which serves as the primary fit observable in the analysis (Table 1).

The following sources of uncertainty are taken into account using constrained nuisance parameters in the fit for all processes. These parameters are used to encode the impact of systematic uncertainties on the expected signal and background yields and shapes. Unless specified otherwise, these are considered correlated across the data taking years. An exception is made for the top quark background. The constrained nuisance parameters included for the top quark background account only for the statistical uncertainties. Systematic uncertainties that impact the top quark background are included in the simultaneous fit to data in the SR and SB through unconstrained bin-by-bin multiplicative parameters, as described in Section. 6. The resulting post-fit uncertainties in these parameters reflect the fit uncertainty in the top quark background prediction.

- *Integrated luminosity*: The integrated luminosities for the 2016, 2017, and 2018 data-taking years have 1.2–2.5% individual uncertainties [64, 65], while the overall uncertainty for the 2016–2018 period is 1.6%.
- $p_T^{miss}$*trigger*: A normalization uncertainty of 1.1% is assigned to all processes, based on the level of agreement observed between data and simulation.
- *Lepton identification and isolation*: A normalization uncertainty is assigned to account for the residual differences between data and simulation in the lepton identification and isolation efficiencies after applying all corrections. In the $\ell \tau _h$ channel, a 0.4% uncertainty is applied for both electron identification and isolation, and 1% (0.1%) for muon identification (isolation), affecting all processes.
- ParticleNet *AK8 *$\hbox {b}\bar{\text {b}}$*-tagging*: A 5% normalization uncertainty is applied to signal AK8 jets to account for the residual data-to-simulation mismodeling of b tagging. This uncertainty reflects the relatively uniform effect of the b tagging uncertainty across the $p_{\textrm{T}}$ spectrum of the AK8 jets.

The following sources of uncertainty affect the shape of the distributions, as well as the normalization of the various backgrounds and the signal, and are applied to all search channels.

- *PDF uncertainties*: Uncertainties from the PDFs are estimated by reweighting the samples with the NNPDF3.0 [66] replicas [67] for the 2016 data set and NNPDF3.1 [42] for the 2017 and 2018 data sets. The variations across the replicas are used to construct an envelope that captures the spread in predictions, which is then taken as the systematic uncertainty.
- *Factorization and renormalization scales*: Variations in the renormalization and factorization scales by factors of 0.5 and 2.0 are considered independently with the exception of simultaneously varying both. Their impact is calculated from the envelope of these variations and included as systematic uncertainties for all processes.
- *Pileup modeling*. Systematic uncertainties from pileup modeling are taken into account by varying the total inelastic cross section used to calculate pileup distributions in simulation by ${\pm }4.6\%$ [68].
- *Jet energy scale and resolution*: Reconstructed jet four-momenta in the simulation are varied according to the uncertainties in the jet energy scale, which are split into 11 different sources. Additionally, the $p_{\textrm{T}}$ of the jets is stochastically changed within the detector resolution. These uncertainties are coherently propagated to all observables, including $p_{\textrm{T}} ^\text {miss}$  [69]. Some of the 11 sources are uncorrelated across the data taking years. The average effect from these sources of uncertainty ranges from 2 to 5%.
- $p_T^{miss}$* mismodeling*: An additional source of uncertainty specific to $p_{\textrm{T}} ^\text {miss}$ arises from unclustered energy, primarily due to calorimeter deposits not associated with any reconstructed object. This unclustered energy is varied within its uncertainties, and the resulting impact is propagated to the $p_{\textrm{T}} ^\text {miss}$.
- *b tagging*: The b tagging and light-flavor misidentification efficiency scale factors, along with their associated uncertainties, are measured using independent control samples [70]. The uncertainties in these scale factors are then propagated to the analysis. Some sources of these uncertainties are uncorrelated across data taking years.
- *Simulation sample size*: Uncertainties due to the limited size of the simulated signal and background samples are included by allowing each bin of the distributions used in the signal extraction to fluctuate independently according to the statistical uncertainties in simulation [71].
- *Uncertainty related to ECAL mistiming*: Partial mistiming of signals in the forward regions of the ECAL endcaps led to a minor reduction in trigger efficiency. Simulations are corrected to emulate the behavior of the data, and the uncertainty in these corrections is propagated to the distributions used in the signal extraction.
- *Electroweak and QCD k-factors*: Uncertainties in the NLO/LO *k*-factors [33–38] calculated for $\text {W}$+jets and $\text {Z}$+jets processes are considered. These uncertainties account for missing higher-order corrections. For QCD, this comes from variations of the factorization and renormalization scales. For electroweak processes, an estimate of the size of the missing higher-order corrections is obtained by taking the difference between applying and not applying the NLO/LO electroweak *k* factors.
- *Hadronically decaying *τ* lepton identification*: Uncertainties in the DeepTau efficiency corrections, for the discrimination of genuine $\uptau _\text {h}$ against jet, electron, and muon are correlated across $\boldsymbol{\uptau }$ decay modes and years for the standard $\boldsymbol{\uptau }$ leptons. Correction factors are derived to account for differences between data and simulation in the identification of boosted $\boldsymbol{\uptau }$ leptons from the BoostedDeepTau algorithm. These were derived using boosted Z boson decays, utilizing events with a spatially close muon–$\boldsymbol{\uptau }$ pair. A simultaneous fit is then performed in the ${\upmu } \uptau _\text {h}$ SR and a dimuon control region, with the latter constraining the normalization of the Drell–Yan process. The data-to-simulation factors were found to be 0.90 per identified boosted $\boldsymbol{\uptau }$ lepton, with an associated uncertainty of 10%. This corresponds to a normalization uncertainty of 20% in the $\tau _h\tau _h$ channel and 10% in the $\ell \tau _h$ channel [55].
- *Hadronically decaying *τ *lepton energy scale*: For the $\uptau _\text {h}$ energy scale contribution to the systematic uncertainty, the four-momenta of reconstructed and identified $\uptau _\text {h}$, including both standard and boosted $\uptau _\text {h}$ objects, are varied by ${\pm } 3\%$ [52]. The resulting variations are propagated to all kinematic observables used in the analysis. For minor backgrounds, this effect is accounted for by a 10% normalization uncertainty, while for the signal a dedicated shape systematic is assigned.

## Results

**Fig. 4:**
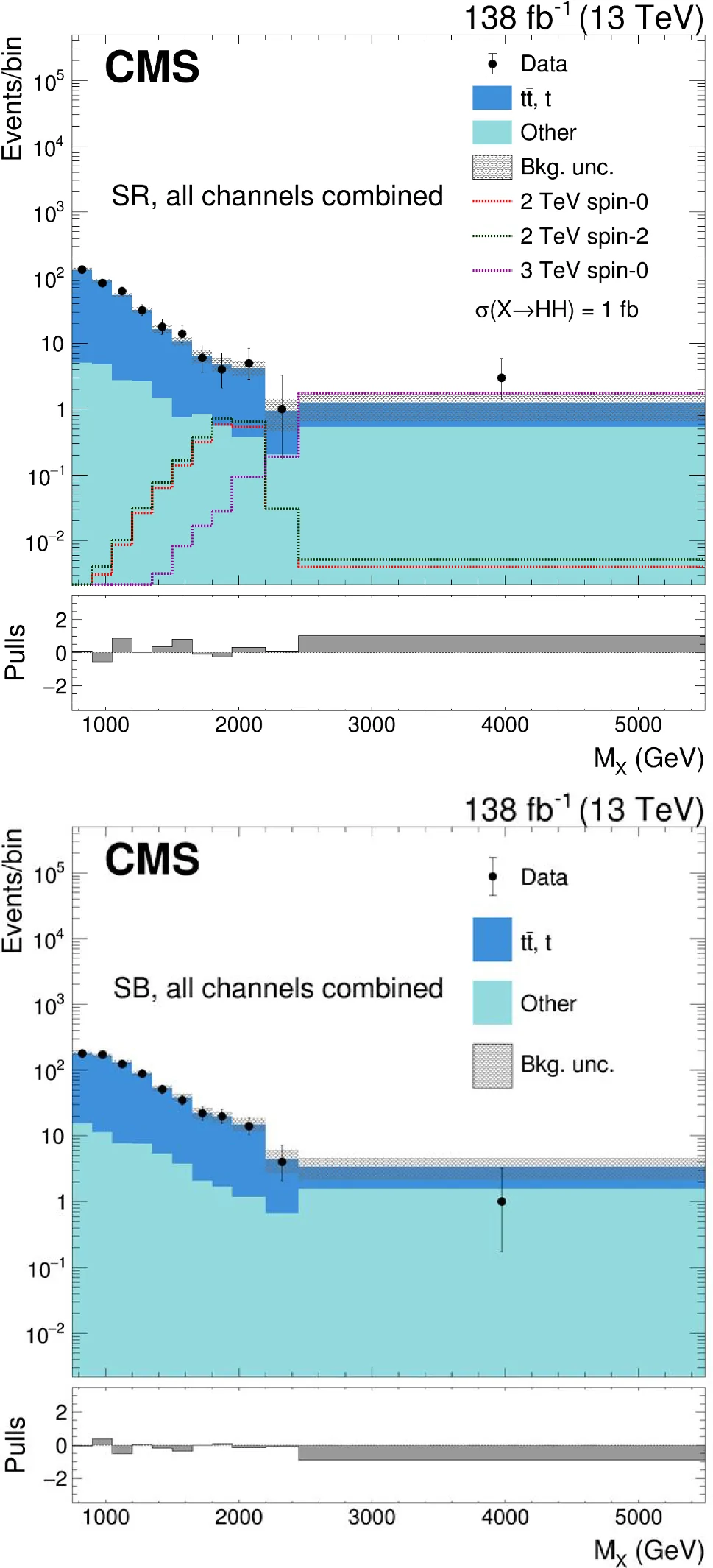
Post-fit reconstructed mass distribution of resonance X in the SR (upper) and SB (lower) after applying all selection criteria, for the sum of the $\tau _h\tau _h$ and $\ell \tau _h$ channels. Minor background contributions are grouped into a single category labeled “Other”. Also shown are several signal predictions in dashed colored lines for visualization only. The lower panels show the “Pull” defined as $\text {(Data - Prediction)/Total uncertainty}$

The analysis follows a blinded strategy, with all aspects of the event selection, background estimation, and statistical fitting procedure defined prior to unblinding data in the SR. The post-fit distributions in $M_{{\text {X}}}$ are shown in Fig. 4 for the SR and SB. In the lower mass region, where the expected background yield is higher, we use a binning comparable to the expected signal width. For resonance masses above $2~\,\text {Te}\hspace{-.08em}\text {V}$, the background event counts become sparse. We therefore merge adjacent bins to avoid empty bins. The content of the final bin also includes events in the overflow region. The data are found to be in agreement with the SM background predictions within the uncertainties. We place upper limits on the production cross section for a heavy resonance X decaying to a pair of Higgs bosons.

**Fig. 5:**
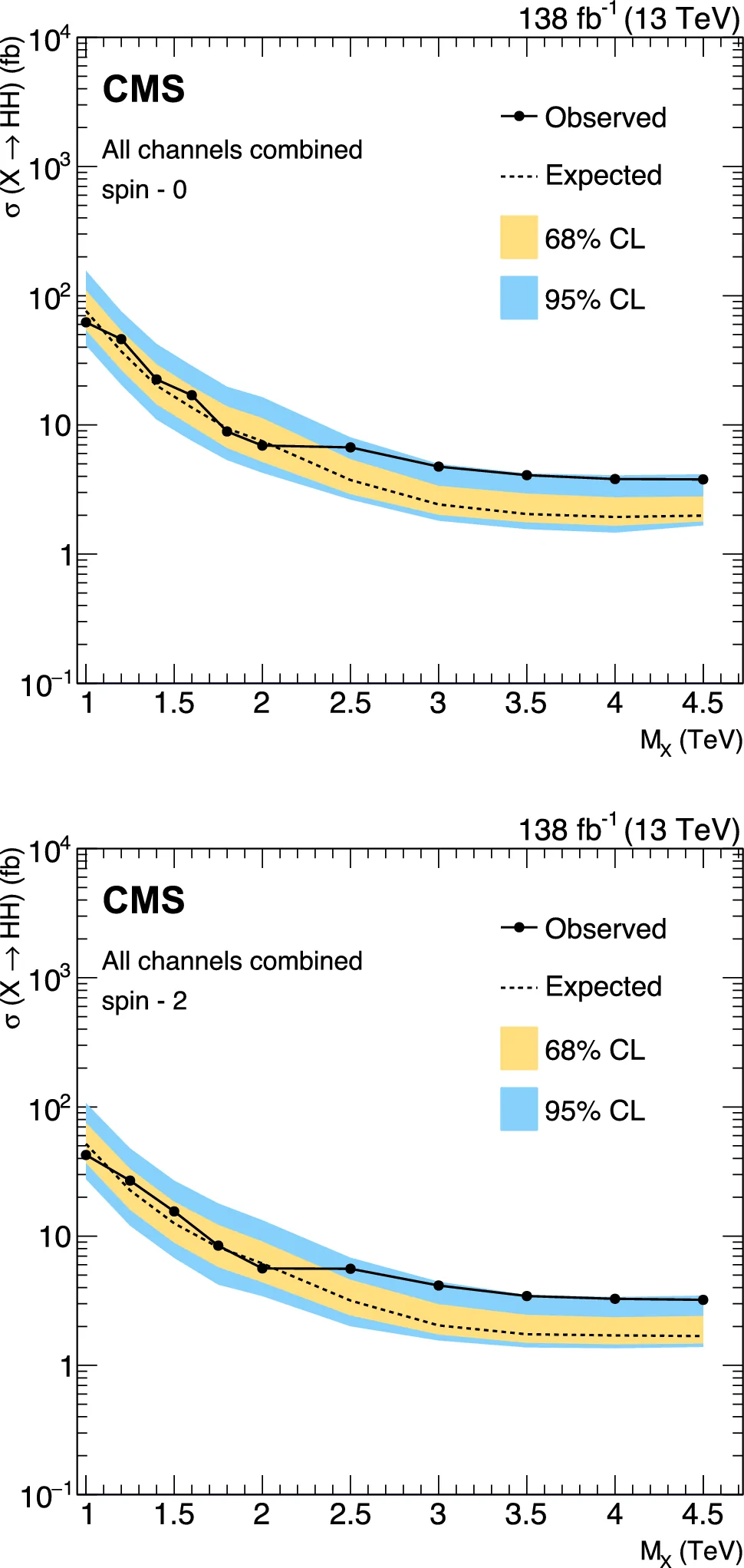
Expected and observed upper limits at 95% CL on the production cross section of resonant HH production for a spin-0 (upper) and spin-2 (lower) narrow resonance. This calculation assumes SM branching fractions of the H boson as discussed in Section 1. The observed limits are higher than the expected limits for mass points above 2.5 TeV, primarily because of the excess of events visible in the highest bin of Fig. 4 (upper)

The 95% $\text {CL}$ upper limits on the ${\text {X}} \rightarrow {\text {HH}}$ production for spin-0 and spin-2 resonances in the mass range from 1 to 4.5$\,\text {Te}\hspace{-.08em}\text {V}$, computed using the modified frequentist $\text {CL}$ $_\text {s}$ method with a profile likelihood ratio test statistic, are shown in Fig. 5. The distributions of the test statistic under the signal-plus-background and background-only hypotheses are obtained from ensembles of pseudo-experiments. The results are scaled by the SM branching fractions of the H bosons and the subsequent decays included in this analysis. The observed (expected) upper limits reach down to 62.2 fb (76.2 fb) for spin-0 and 42.5 fb (51.8 fb) for spin-2 at 1$\,\text {Te}\hspace{-.08em}\text {V}$. At 4.5$\,\text {Te}\hspace{-.08em}\text {V}$, the observed (expected) upper limits are 3.8 fb (2 fb) for spin-0 and 3.2 fb (1.7 fb) for spin-2. The results for spin-2 are broadly similar to those for spin-0. However, the $p_{\textrm{T}} ^\text {miss}$ spectrum for spin-2 signals is shifted toward higher values, which slightly increases the signal efficiency. This effect is visible in the superimposed signal templates for spin-0 and spin-2 in Fig. 4 and is reflected in the slightly stronger expected limits for the spin-2 scenario. The observed limits are higher than the expected limits for mass points above 2.5 TeV, primarily because of the excess of events visible in the highest bin of Fig. 4 (upper).

This analysis improves upon the spin-0 resolved ATLAS search [15] for resonance masses above 1.4$\,\text {Te}\hspace{-.08em}\text {V}$. Furthermore, it is significantly better than the spin-0 search from ATLAS in the Lorentz-boosted regime [14], covering the 1–3$\,\text {Te}\hspace{-.08em}\text {V}$   mass range, across the entire mass range considered in that analysis. The results presented in this paper represent the most sensitive upper limits to date in the resonance mass range of 1.4–4.5$\,\text {Te}\hspace{-.08em}\text {V}$   in the $\hbox {b}\bar{\text {b}}\,\tau ^{+}\tau ^{-}$ final state.

## Summary

A search has been presented for heavy resonant Higgs boson pair (HH) production in the $\hbox {b}\bar{\text {b}}\,\tau ^{+}\tau ^{-}$ final state, exploring resonance masses between 1 and 4.5$\,\text {Te}\hspace{-.08em}\text {V}$. The analysis is based on pp   collision data collected with the CMS detector during 2016–2018, corresponding to an integrated luminosity of 138$\,\text {fb}^{-1}$ at a center-of-mass energy of 13$\,\text {Te}\hspace{-.08em}\text {V}$. In this mass regime, the H bosons produced are boosted sufficiently to result in collimated decay products. The reconstruction and identification of such boosted objects are enhanced using advanced machine learning techniques, including a graph convolutional neural network for merged $\hbox {b}\bar{\text {b}}$ jets and a convolutional neural network for boosted $\tau ^{+}\tau ^{-}$ identification.

No significant deviation from the standard model background expectation is observed, and 95% confidence level upper limits are set on the production cross section of a heavy resonance decaying to HH, evaluated independently for both spin-0 and spin-2 hypotheses. This analysis sets the most sensitive upper bounds to date on the production of ${\text {X}} \rightarrow {\text {HH}} \rightarrow \hbox {b}\bar{\text {b}}\,\tau ^{+}\tau ^{-}$ in the mass range of 1.4–4.5$\,\text {Te}\hspace{-.08em}\text {V}$.

## Data Availability

Release and preservation of data used by the CMS Collaboration as the basis for publications is guided by the CMS data preservation, re-use and open access policy.

## Code Availability Statement

This manuscript has associated code/software in a data repository. [Author’s comment: The CMScore software is publicly available on GitHub (https://github.com/cms-sw/cmssw).]
